# Compulsivity is linked to reduced adolescent development of goal-directed control and frontostriatal functional connectivity

**DOI:** 10.1073/pnas.1922273117

**Published:** 2020-09-28

**Authors:** Matilde M. Vaghi, Michael Moutoussis, František Váša, Rogier A. Kievit, Tobias U. Hauser, Petra E. Vértes, Nitzan Shahar, Rafael Romero-Garcia, Manfred G. Kitzbichler, Edward T. Bullmore, NSPN Consortium, Raymond J. Dolan

**Affiliations:** ^a^Max Planck University College London Centre for Computational Psychiatry and Ageing Research, University College London, WC1 B5EH London, United Kingdom;; ^b^Wellcome Centre for Human Neuroimaging, University College London, WC1N 3AR London, United Kingdom;; ^c^Department of Psychiatry, University of Cambridge, CB2 2QQ Cambridge, United Kingdom;; ^d^Centre for Neuroimaging Sciences, Institute of Psychiatry, Psychology & Neuroscience, King’s College London, SE5 8AF London, United Kingdom;; ^e^Medical Research Council Cognition and Brain Sciences Unit, Cambridge, CB2 7EF Cambridge, United Kingdom;; ^f^The Alan Turing Institute, NW1 2DB London, United Kingdom;; ^g^School of Mathematical Sciences, Queen Mary University of London, E1 4NS London, United Kingdom

**Keywords:** adolescence, development, model-based control, compulsivity, frontostriatal connectivity

## Abstract

A characteristic of adaptive behavior is its goal-directed nature. An ability to act in a goal-directed manner is progressively refined during development, but this refinement can be impacted by the emergence of psychiatric disorders. Disorders of compulsivity have been framed computationally as a deficit in model-based control, and have been linked also to abnormal frontostriatal connectivity. However, the developmental trajectory of model-based control, including an interplay between its maturation and an emergence of compulsivity, has not been characterized. Availing of a large sample of healthy adolescents (*n* = 569) aged 14 to 24 y, we show behaviorally that over the course of adolescence there is a within-person increase in model-based control, and this is more pronounced in younger participants. Using a bivariate latent change score model, we provide evidence that the presence of higher compulsivity traits is associated with an atypical profile of this developmental maturation in model-based control. Resting-state fMRI data from a subset of the behaviorally assessed subjects (*n* = 230) revealed that compulsivity is associated with a less pronounced change of within-subject developmental remodeling of functional connectivity, specifically between the striatum and a frontoparietal network. Thus, in an otherwise clinically healthy population sample, in early development, individual differences in compulsivity are linked to the developmental trajectory of model-based control and a remodeling of frontostriatal connectivity.

## Significance

Goal-directed behavior is impaired in disorders of compulsivity. Here, we characterize the developmental trajectory of model-based control and show a progressive strengthening from adolescence to early adulthood. We found that the presence of compulsivity traits impacts on this trajectory as well as on the degree of remodeling in functional connectivity within frontostriatal circuits. These findings have implications for understanding the interplay between compulsivity, the developmental trajectory of model-based planning, and functional connectivity in frontostriatal circuits.

Adaptive behavior often entails choices that are goal-directed, mediated by a rich representation of prospective outcomes and supported by a cognitive model of the environment. Alternatively, choices can be habitual where, through prior repetition and learning, they can be executed without deliberation (1, 2). The distinction between the goal-directed and the habitual control system of decision making is well described in psychology and neuroscience (1, 3–7). More recently, computational reinforcement learning models have formalized these two behavioral strategies in terms of model-based and model-free control, respectively (8, 9).

A body of evidence supports the notion that decision making in adults is guided by an interaction between these two manifestations of instrumental behavioral control (3, 4, 9). Importantly, a fine-tuning in this balance may be subject to refinement during important developmental periods, such as adolescence, and to destabilization in the context of psychiatric disorders. Developmental studies have begun to characterize the typical trajectory of model-based control in young children as weakly, or less readily, deployed (10, 11). However, an ability to deploy model-based control strengthens over the course of adolescence and early adulthood (10). Adolescent development is also characterized by neural reorganization, particularly involving areas implicated in high-order cognitive functions (12–15), such as model-based control (9). The transition into adulthood is characterized by a decrease in subcortical–cortical connectivity and strengthening of cortico–cortical connectivity, particularly within association cortices (12–15). More recently, an analysis of resting-state functional connectivity in the sample investigated here indicated that subcortical structures have the greatest magnitude of functional connectivity reorganization, whereby connections that were strong at 14 y of age became weaker during the course of adolescent development (16).

Importantly, model-based control can be compromised in specific psychiatric disorders where behaviors are repetitive, maladaptive, and out of control (17–21). For example, obsessive-compulsive disorder (OCD) is characterized by repetitive unwanted actions and thoughts (American Psychiatric Association, *DSM-5*) (22), possibly reflecting an imbalance between model-based and model-free control (17). More generally, the construct of “compulsivity” has been accounted for in terms of an aberrantly weak “goal-directed” system or, equivalently, compromised model-based control (23, 24).

Animal experiments indicate that goal-directedness is supported by a frontostriatal circuitry (6). In line with these findings, disruption of the dorsolateral prefrontal cortex (DLPFC) in healthy humans impairs an ability to deploy model-based control (25), while an associated frontostriatal network (including the DLPFC) is dysfunctional in patients with compulsivity disorders (26). Abnormalities of frontostriatal circuitry in OCD are evident under cognitive demands that tax goal-directed control (27–30), and in resting-state functional connectivity measures (31, 32), with evidence of a relation of the latter to goal-directed cognitive performance (33).

However, several questions remain unanswered. A cross-sectional approach adopted in extant studies limits the inferences regarding a temporal interplay between the development of model-based control, the maturation of frontostriatal circuits, and the emergence of compulsivity. For example, it remains untested whether model-based control is informative as regards to the trajectory of individual differences in compulsivity over time and, vice versa, whether the presence of compulsivity is linked to the developmental trajectory of model-based control.

To investigate the temporal relationship between the clinical domain of compulsivity and the computational domain of model-based control, we utilized data from an accelerated longitudinal study of healthy adolescents (*n* = 569; aged 14 to 24 y). On at least two occasions, ∼18-mo apart, all participants completed a classic two-step reinforcement decision task, widely used to quantify differences in model-based control (9), as well as standard questionnaires measuring individual differences in compulsivity.

We extend on previous findings indicating a consolidation of model-based control during adolescence (10), by now showing a within-subject longitudinal increase in model-based control, where the rate of individual improvement in model-based control is more pronounced for younger subjects. Second, using a bivariate latent change score model (34, 35), we show that model-based control is less pronounced in the presence of high compulsivity traits, and that a within-subject developmental strengthening in model-based control is conditioned by individual variability in compulsivity traits. Finally, using resting-state functional MRI (fMRI) data we demonstrate that a developmental trajectory of frontostriatal connectivity is moderated by the presence of compulsivity during adolescence, such that within-subject developmental changes in frontostriatal connectivity are less pronounced in subjects with high compulsivity.

## Results

### Sample.

**Fig. 1.**
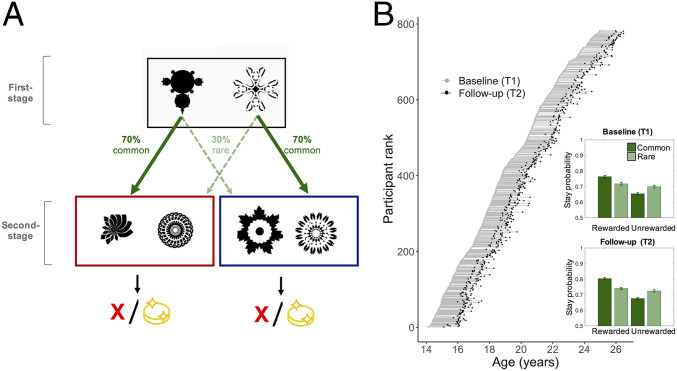
Experimental task and procedure. (*A*) Schematic of the two-step reinforcement learning task used to assess model-based control. In the first stage, participants have to choose between two fractals, and this probabilistically determines the transition to red or blue state at the second stage. For example, the fractal on the left conferred a 70% chance of transition to the red state in the second stage (“common” transition) and a 30% chance of transition to the blue state in the second stage state (“rare” transition). These transition probabilities were fixed and could be learned over time. At the second stage state, participants have to choose between the two available fractals, each of which is associated with a distinct probability of being rewarded (signified by a coin). The probability of receiving a reward associated with each second stage fractal was not fixed (unlike the transition structure) but drifted slowly over time (0.2 < *P* < 0.8). Better model-based control is indexed by an ability to take account of the reward history as well as the transition structure. For example, if a first-stage choice is followed by a rare transition to the second stage, and the second-stage choice results in reward, a “model-based” participant would be more likely to choose the alternative first-stage choice on the next trial as doing so is predicted to lead to the common transition and the just experienced reward at the second stage. (*B*) Participants were assessed in an accelerated longitudinal design. Each unique participant is represented by a different row, ordered by age at first assessment. Connecting lines join repeated cognitive measurements from the same participant (baseline, T1; follow-up, T2); 569 participants completed the two-step reinforcement task at both time points. As expected, data illustrate that participants used a mixture of model-based and model-free learning to guide choice both at baseline (T1, *Upper Inset*) and follow-up (T2, *Lower Inset*), consistent with previous studies. Model-based and model-free decision parameters estimated by the logistic regression analysis are shown in *SI Appendix*, Table S1. Error bars show SEM.

We studied a large sample of adolescents (*n* = 569; 280 females, aged 14 to 24 y), within an accelerated longitudinal design encompassing a time window sensitive to developmental change. At two distinct time points (T1, baseline; T2, follow-up), ∼18-mo apart, participants were assessed on a reinforcement learning task (*Materials and Methods* and Fig. 1*A*) commonly used to quantify individual differences in model-based control (9). At temporal proximity to our experimental sessions (*Materials and Methods*), they also completed self-reported questionnaires assessing individual differences in compulsivity. We also analyzed resting-state measures of frontostriatal functional connectivity obtained via MRI, derived from a subsample (*n* = 230) who partook in our experimental cognitive assessment sessions. Most of participants (*n* = 178) in the fMRI cohort were scanned twice, at temporal proximity to our experimental sessions (*Materials and Methods*); 52 participants were scanned once. For details on cohort selection, behavioral and imaging preprocessing, and quality control, see *Materials and Methods*.

### Development of Model-Based Control.

For each assessment and for each participant, we first quantified individual differences in model-based control. Model-based control was operationalized as a parameter estimate from a logistic regression analysis predicting choices during iterations of the task (see *Materials and Methods*). A model-based control strategy is indexed by an interaction between reward and transition structure on behavior, where an agent is more likely to repeat a rewarded action if the transition is common. In contrast, a model-free influence is mirrored by a main effect of reward, whereby an agent exhibits sensitivity to whether or not the trial was rewarded alone, and does not modify the behavior as a function of the underlying transition structure (Fig. 1*A*). In this way, we identified a behavioral signature of both model-free and model-based control at each time point, indexed by a significant main effect of reward and a significant interaction between reward and transition type, respectively, replicating previous findings (9) (Fig. 1 *B*, *Inset*, and *SI Appendix*, Table S1).

To investigate the maturational trajectory of model-based control, we used these estimates of model-based control, computed separately at each assessment, as dependent variables in a linear mixed-effects (LME) model. This model was informed by analysis recommendations (36) successfully adopted in recent studies (37, 38) (*Materials and Methods* and *SI Appendix*). This tested jointly how model-based control varies with mean age of subjects (i.e., age mean, between-subject effect of age) and how it changes over time (i.e., visits/time, within-subject effect of age), as well as their interaction (*Materials and Methods* and *SI Appendix*, Table S2). The latter allowed us to ask how within-subject changes in model-based control depend on the mean age of a subject, regardless of other covariates included in the model (i.e., IQ and gender).

**Fig. 2.**
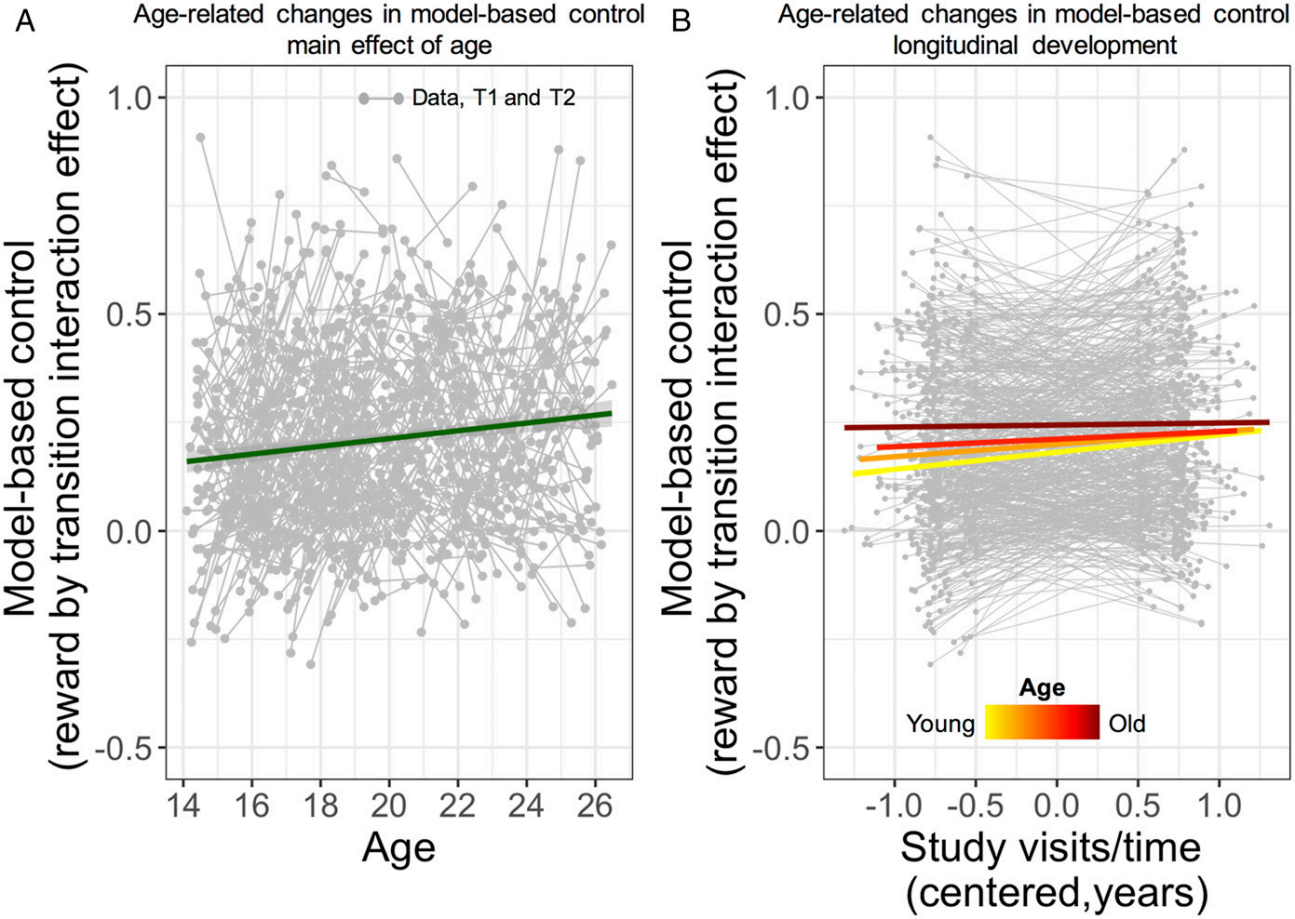
Age-related (between-subject) and developmental (within-subject) changes in model-based control. (*A*) Model-based control increases over the course of adolescence as a function of age. The gray points and connecting lines represent the paired (T1, baseline and T2, follow-up) assessments for all participants and the fitted line is the main effect of mean age from the LME model (i.e., assuming major trend is linear). On the *x* axis, age refers to age of each individual at each time point. (*B*) Developmental rate of change in model-based control is more pronounced in subjects with younger mean age than subjects with older mean age. Colored lines illustrate the significant interaction between mean age and visits/time, which indicates that the developmental rate of increase in model-based control was dependent on mean age of the subjects (yellow to red coloration for younger and older adolescents respectively). *A* and *B* display a main effect of age and an interaction between mean age and time/visits on model-based control from a regression model with the following effects of interest: Intercept, visits/time, gender, IQ, age mean, gender by visits/time, IQ by visits/time; age mean by visits/time (see also *SI Appendix*). T1, baseline; T2, follow-up.

Consistent with previous findings (10), we identified a between-subject effect of age such that older participants had better model-based control (β = 0.006, SE = 0.002, df = 537, *t* = 2.28; *P* = 0.023) (Fig. 2*A* and *SI Appendix*, Table S2). We also found a within-subject effect of age, indicating longitudinal development of model-based control, evident in a significant increase of model-based control at follow-up (β = 0.024, SE = 0.009, df = 537, *t* = 2.63; *P =* 0.009) (Fig. 2*B* and *SI Appendix*, Table S2). Strikingly, this rate of improvement was more pronounced in the youngest participants, as shown by a significant interaction between visits/time and subject mean age (β = −0.005, SE = 0.002, df = 537, *t* = −2.26; *P* = 0.024) (Fig. 2*B* and *SI Appendix*, Table S2). These findings highlight that model-based control undergoes significant change in the transition from adolescence into early adulthood, possibly reaching a plateau in late adolescence beyond which no further appreciable change is observed.

Higher IQ was associated with better model-based control (β = 0.052, SE = 0.008, df = 537, *t* = 6.750 *P* < 0.001) and males were more model-based relative to females (β = 0.035, SE = 0.014, df = 537, *t* = 2.46; *P* = 0.014). Importantly, the within-subject rate of development in model-based control was independent of both these factors (*SI Appendix*, Table S2). To investigate the specificity of these developmental changes in model-based control, we used the same analytical approach to examine the maturational trajectory of an alternative, less-sophisticated, model-free strategy (*SI Appendix*, Table S3). Here, there was a between-subject effect of age (β = 0.011, SE = 0.003, df = 537, *t* = 4.07; *P* < 0.001), while a within-subject effect of age was nominally not significant (β = 0.020, SE = 0.010, df = 537, *t* = 1.95; *P* = 0.051). Crucially, the within-subject rate of model-free change was not influenced by a participant’s mean age (β = −0.003, SE = 0.002, df = 537, *t* = −1.19, *P* = 0.235).

### Testing for Training Versus Developmental Effects on Model-Based Control.

In follow-up analyses, we asked whether longitudinal changes might be explained simply as retest effects (i.e., familiarity with the task or practice effect at follow-up might cause greater use of model-based strategies for task performance) rather than developmental effects. To provide a principled basis to assess a retest effect, we focused on data from a subsample of participants who were tested 6 mo after (“retest” sample, T1R) the first assessment (T1), in addition to the follow-up time-point (T2). We then used logistic regression on data from participants who came to the laboratory for the T1, the T1R (i.e., 6 mo), and the T2 (i.e., 18 mo) assessments (*n* = 53) (*Materials and Methods* and *SI Appendix*, Table S5). This analysis, which included IQ, age, and gender as fixed covariates, showed a significant reward-by-transition-by-session interaction for the T2 follow-up visit (β = 0.127, SE = 0.062, *z*-value = 2.06, *P* = 0.039) but critically not for the retest T1R visit (β = 0.006, SE = 0.071, *z*-value = 0.09, *P* = 0.933). These results provide no support for a mere training or repetition effect, as the expectation would be for a greater change after 6 mo than after 18 mo. This was not the case, either numerically nor inferentially.

### High Compulsivity Is Associated with Decreased Model-Based Control.

We next tested the relationship between individual differences in compulsivity and model-based control. As predicted, we found a significant association such that greater expression of compulsivity traits correlated with poorer model-based control at T1 (Pearson’s correlation *r* = −0.11, *t* = −2.63, df = 535, *P* = 0.009) and T2 (Pearson’s correlation *r* = −0.18, *t* = −4.15, df = 532, *P* < 0.001). These findings were confirmed in a logistic regression analysis, where we included compulsivity as a between-subjects predictor and tested for interactions with all other factors in the model (i.e., age, gender, and IQ were included as fixed-effects predictors) (*SI Appendix*, Table S6). The three-way interaction between reward, transition type, and compulsivity showed that model-based control was less marked in those with higher levels of compulsivity (β = −0.034, SE = 0.009, *z*-value = −3.81, *P* < 0.001). The effect of age on model-based control was no longer significant in this analysis, which included data from T1 and T2 (β = 0.021, SE = 0.012, *z*-value = 1.78, *P* = 0.075). This is likely due to a diluted effect of age when collapsing data from both time-points, as the role of age of model-based control was likely weaker at T2 when participants had already reached a more advanced maturational stage (Fig. 2).

### High Compulsivity Is Associated with Reduced Developmental Increase in Model-Based Control.

Having identified a developmental change in model-based control, and an association between model-based control and compulsivity, we next probed their reciprocal influences over time. For this we employed a latent change score model (34, 35), testing a hypothesis that individual differences in compulsivity are associated with distinct developmental trajectories in model-based control. This model also allowed us to test a reciprocal hypothesis that individual differences in model-based control predict rate of change in compulsivity. In other words, we examined the extent to which longitudinal change in one domain is influenced by the starting level in another domain.

**Fig. 3.**
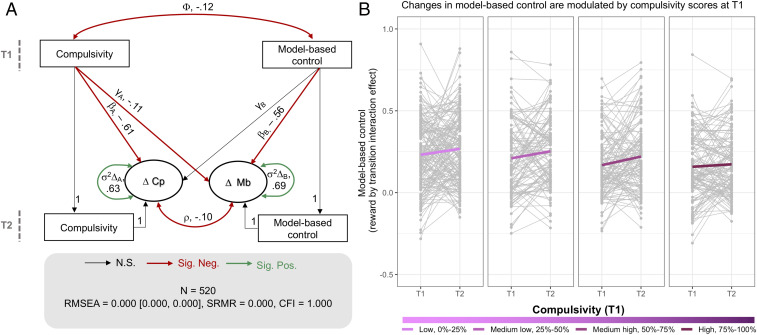
Relationship between compulsivity and within-subject change in model-based control. (*A*) Bivariate latent change score model. Circles indicate latent variables, and rectangles indicate observed variables. Single-headed arrows indicate regressions; double-headed arrows indicate variances and covariances. Key parameters are indicated by letters (ϕ, covariance between compulsivity and model-based control at baseline; mechanisms of change: γ, effect of coupling; γ_A_ compulsivity → rate of change of model-based control; γ_B_, model-based control → rate of change of compulsivity; β, self-feedback effect for compulsivity (β_A_) and model-based control (β_B_); dynamics of change: ρ, change covariance; σ variance in the rate of change for compulsivity (σ^2^Δ_A_) and model-based control (σ^2^Δ_B_)). A “1” indicates which values were constrained to unity. Values are standardized parameter estimates. Significant parameter estimates are shown in red (negative associations) and green (positive associations) bolded lines. (*B*) Raw data for change in model-based control from T1 to T2 plotted as a function of compulsivity at T1. The raw scores are plotted on separate panels for different groups of subjects, classified based on their compulsivity at T1, but the model was estimated for the population as a whole. For illustration purposes only, the subjects were divided into four groups based on first (low, 0 to 25%), second (medium low, 25 to 50%), third (medium high, 50 to 75%), and fourth (high, 75 to 100%) quantile of the distribution of compulsivity at T1. Lines illustrate within-subject changes dependent on different compulsivity scores at T1 (pink to purple coloration for lower to higher compulsivity respectively). There wasn’t a significant difference in age between the four groups (see text; low compulsivity, mean age: 18.83 ± 3.1, range: 14.28 to 24.98; medium low compulsivity, mean age T1: 19.28 ± 3.09, range: 14.2 to 24.92; medium high compulsivity, mean age T1: 18.61 ± 2.68, range: 14.43 to 24.35; high compulsivity, mean age T1: 18.8 ± 2.93, range: 14.1 to 24.83, see main text). Cp, compulsivity; Mb, model-based control; N.S., not significant; Sig. Neg. significant negative pathway; Sig. Pos. significant positive pathway; T1, baseline; T2, follow-up. Indexes of model fit are reported for consistency but in the context of a fully saturated model (as the one reported) they should not be interpreted as evidence of good fit.

We found, as expected (Fig. 3 and *SI Appendix*, Table S7), a significant negative correlation between the two domains at T1 (Fig. 3*A* and *SI Appendix*, Table S7) (*z*-value = −2.797, *P* = 0.005, standardized estimate = −0.12) such that higher levels of compulsivity were associated with reduced model-based control. Additionally, there were within-subjects differences in the rate of change in compulsivity (*z*-value = 9.557, *P* < 0.001, standardized estimate = 0.63) and model-based control (*z*-value = 16.775, *P* < 0.001, standardized estimate = 0.69), as indicated by the significance associated with the respective variances in the rate of change (Fig. 3*A*).

Interestingly, the model accounted for the association between the two domains at T1 and showed that compulsivity at T1 influenced the rate of developmental change of model-based control. Specifically, high compulsivity levels at baseline had an effect on within-subject change in model-based control (*z*-value = −3.131, *P* = 0.002, standardized estimate = −0.11) (Fig. 3*A*), indicating that high compulsivity was linked to reduced strengthening of model-based control over time (Fig. 3*B*). Convergently, removal of the path linking compulsivity to within-subject change in model-based control resulted in a significant deterioration in model fit (Δχ_2_ = 9.597, df = 1, *P* = 0.002), suggesting a model where this pathway was included was preferred. In contrast, model-based control at T1 was not associated with within-subject change in compulsivity (*z*-value = −0.314, *P* = 0.754, standardized estimate = −0.010). Finally, above and beyond the coupling parameters, the rates of change were still, weakly, negatively correlated (*z*-value = −2.593, *P* = 0.010, standardized estimate = −0.103), indicative of less change in model-based control for those who changed the most in terms of compulsivity. This finding highlights a possibility of other, unmeasured, mechanisms driving both rates of change.

These results were unchanged when age, gender, and IQ were regressed on the observed variables at T1 and on the latent change variables of both model-based control and compulsivity (*SI Appendix*, Table S8). In this model, compulsivity influenced the developmental trajectory of model-based control, while accounting for potential sources of shared variance due to a baseline association between age and model-based control. There were no differences in mean age at T1 for groups with different compulsivity scores, defined based on quantiles of the compulsivity distribution [*F*(3, 516) = 1.059, *P* = 0.366, post hoc comparison all *P*s > 0.312]. Similar findings were obtained using a secondary measure of compulsivity, the Padua Inventory Washington University Revision (PI-WSUR), available at both time points for a smaller subset of participants (*Materials and Methods* and *SI Appendix*).

Finally, our findings were specific, as shown when using model-free rather than model-based control scores in our model. While this model provided a good fit to data (*n* = 520; χ^2^ = 0.288, df = 1, *P* = 0.591; root-mean-square error of approximation [RMSEA] = 0.000 [0.000, 0.094], standardized root mean square residual [SRMR] = 0.004, comparative fit index [CFI] = 1.000, Yuan–Bentler scaling correction factor = 1.001), the individual differences in compulsivity did not predict rate of change in model-free scores over time (*z*-value = −0.814, *P* = 0.416, standardized estimate = −0.026). Removal of the path linking compulsivity to rate of change in model-free control did not compromise model fit (Δχ^2^ = 0.657, df = 1, *P* = 0.418), indicating a more parsimonious model, with no direct path between compulsivity at baseline and within-subject change in model-free control, was preferred.

### High Compulsivity Is Associated with Reduced Developmental Changes in Striatal Connectivity.

To establish how an influence of compulsivity on model-based control relates to frontostriatal functional connectivity, we used resting-state data from the fMRI cohort (*Materials and Methods*). We focused on a region of striatum, corresponding to the central lateral zone, shown previously to be preferentially coupled to a frontoparietal network (FPN) (39, 40). A specific focus on connectivity within this circuit was motivated by evidence that compulsivity affects myelination within regions of the FPN in this same sample (37), and by robust and independent evidence showing functional aberrations within this network in OCD (26, 29, 32, 33). In addition, neuroimaging studies in healthy subjects show that activation of brain areas encompassing these regions is associated with a neural signature of model-based behavior (9, 25).

**Fig. 4.**
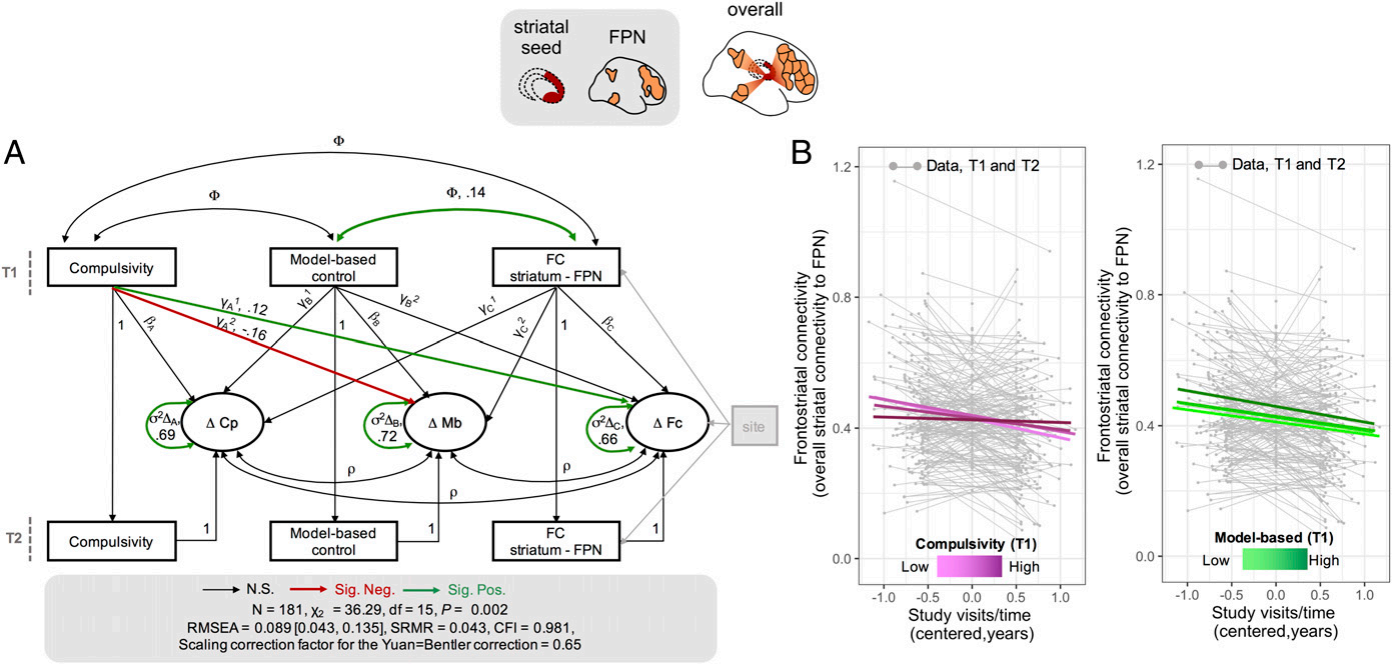
Relationship between compulsivity, model-based control, and within-subject change in frontostriatal connectivity. (*A*) Extended latent change score model. Overall striatal connectivity strength was estimated by averaging the pair-wise correlations between the specific striatal seed and the cortical regions within the FPN. Circles indicate latent variables, and rectangles indicate observed variables. Single-headed arrows indicate regressions; double-headed arrows indicate variances and covariances. Key parameters are indicated by letters (ϕ, covariance at T1; mechanisms of change: γ effect of coupling, γ_A_^1^, compulsivity → rate of change of overall striatal connectivity; γ_A_^2^, compulsivity → rate of change of model-based control; γ_B_^1^, model-based control → rate of change of compulsivity; γ_B_^2^, model-based control → rate of change of overall striatal connectivity; γ_c_^1^, overall striatal connectivity → rate of change of compulsivity; γ_C_^2^, overall striatal connectivity → rate of change of model-based control; β, self-feedback effect for compulsivity [β_A_], model-based control [β_B_], and overall striatal connectivity [β_c_]; dynamics of change: ρ, change covariance; σ^2^ variance in the rate of change for compulsivity [σ^2^Δ_A_], model-based control [σ^2^Δ_B_], and overall striatal connectivity [σ^2^Δ_C_]). *SI Appendix*, Table S9 for precise characterization of the model). (*B*) LME model where compulsivity and model-based control scores at T1 were used to predict rate of within-subject change of overall striatal connectivity, which included site, age, gender, and IQ as fixed covariates. Lines on the panels illustrate the interaction between compulsivity (*Left*) and model-based control (*Right*) at baseline and within-subject rate of change in striatal connectivity to the FPN. Compulsivity, but not model-based control, affects the within-subject change of striatal connectivity with the FPN (see also main text). This indicates that the rate of within-subject change in striatal connectivity to the FPN was dependent on individual differences in compulsivity at baseline. Top schematic shows measures of functional connectivity used. Cp, compulsivity; Mb, model-based control; FC, functional connectivity striatum – FPN; N.S., not significant; Sig. Neg. significant negative pathway; Sig. Pos. significant positive pathway; T1, baseline; T2, follow-up.

For the selected striatal region, we computed an overall striatal connectivity strength, consisting of the average pair-wise correlations between this striatal region and all the cortical regions in the FPN (*Materials and Methods*). We extended our latent change score model to include the overall striatal connectivity strength at T1 and T2 (Fig. 4 and *SI Appendix*, Table S9), allowing us to examine reciprocal interactions between compulsivity, model-based control, and overall striatal connectivity strength. Site was regressed on connectivity measures to account for differences in scanning sites. Using this approach, we tested cross-domain coupling pathways to capture the extent to which within-subject changes in one domain (e.g., overall striatal connectivity) were a function of a baseline level in the other domains (e.g., model-based control or compulsivity) (Fig. 4*A*).

The extended latent change score model provided a good fit to the data (Fig. 4*A*). In line with our simpler implementation in the larger behavioral cohort, we found that individual differences in compulsivity related to the rate of longitudinal change in model-based control (*z*-value = −2.603, *P* = 0.009, standardized estimate = −0.159). Additionally, this model also revealed that individual differences in compulsivity influenced the rate of developmental change in connectivity between the striatum and FPN cortical regions (*z*-value = 2.107, *P* = 0.035, standardized estimate = 0.124), such that higher compulsivity at baseline was predictive of lower rates of within-subject change in striatal connectivity (Fig. 4*A*). In contrast, model-based control did not affect the rate of within-subject change in overall striatal connectivity strength (*z*-value = 0.830, *P* = 0.406, standardized estimate = 0.052) (*SI Appendix*, Table S9). Model fit was significantly decreased when the path linking compulsivity to change in model-based control was removed (Δχ_2_= 6.773, df = 1, *P* = 0.009), as in the behavioral cohort. Model fit significantly deteriorated following deletion of the path linking compulsivity to change in overall striatal connectivity strength (Δχ_2_= 4.309, df = 1, *P* = 0.036). Therefore, models including these pathways were preferable.

The relationship between compulsivity and longitudinal changes in frontostriatal connectivity was confirmed by an additional, independent model, using model-free rather than model-based control scores. Accordingly, individual differences in compulsivity influenced the rate of developmental change in connectivity between the striatum and FPN cortical regions (*z*-value = 2.055, *P* = 0.040, standardized estimate = 0.119), with a significant deterioration of model fit when this path was removed (Δχ_2_ = 4.287, df = 1, *P* = 0.038). Model-free control did not impact the rate of within-subject change in overall striatal connectivity strength (*z*-value = −0.170, *P* = 0.865, standardized estimate = −0.011). Therefore, neither model-based nor model-free scores affected the rate of within-subject change in overall striatal connectivity strength.

Next, we estimated a separate model which, in addition to site, also regressed age, gender, and IQ on the observed variables at T1 and on the latent change variables of model-based control, compulsivity, and overall striatal functional connectivity. The associations between compulsivity and rate of change in model-based control and overall striatal connectivity strength were in the same direction and of similar magnitude to effects detected in the simpler model, albeit not nominally significant, possibly due to an increased complexity of the model (*SI Appendix*, Table S10). In addition, there was evidence that overall striatal connectivity strength influenced the rate of change in compulsivity (*SI Appendix*, Table S10). However, this last finding was detected only with this specific set of covariates and not supported by an alternative analytical approach (see below).

We used an LME model, which systematically account for site, age, gender and IQ (*Materials and Methods* and *SI Appendix*) and observed a within-subject longitudinal decrease in connectivity between the striatum and a FPN (β = −0.057, SD = 0.016, df = 173, *t* = −3.477, *P* = 0.001). The between-subject, cross-sectional, effect of age revealed that older participants had increased connectivity between the striatum and the FPN (β = 0.007, SD = 0.003, df = 224, *t* = 2.169, *P* = 0.031). Consistent with our previous model (illustrated in Fig. 4*A*), compulsivity (β = 0.005, SD = 0.002, df = 173, *t* = 2.108, *P* = 0.037) but not model-based control (β = −0.012, SD = 0.047, df = 173, *t* = −0.259, *P* = 0.799) affected the rate of within-subject change of overall striatal connectivity strength (Fig. 4*B*). In line with previous results (Fig. 4*A*), overall striatal connectivity strength at T1 did not predict the rate of within-subject change in compulsivity (β = 0.16, SD = 1.242, df = 173, *t* = 0.128, *P* = 0.897). To assess the specificity of these findings, we repeated this analysis using the overall connectivity strength between the same striatal seed and a different set of cortical regions belonging to a motor network (40). We did not observe an effect of compulsivity on rate of change of striatal connectivity with cortical regions within a motor network (all *t*s > 1.145, all *P* > 0.254). Finally, our results were robust in so far as the effect of compulsivity on overall striatal connectivity changes was detected even when we used an alternative compulsivity measure, akin to that previously developed to investigate the relationship between compulsivity and brain markers of myelin development (37) (*Materials and Methods* and *SI Appendix*).

### High Compulsivity Affects Connectivity between Striatum and a Specific Set of Frontal Regions.

**Fig. 5.**
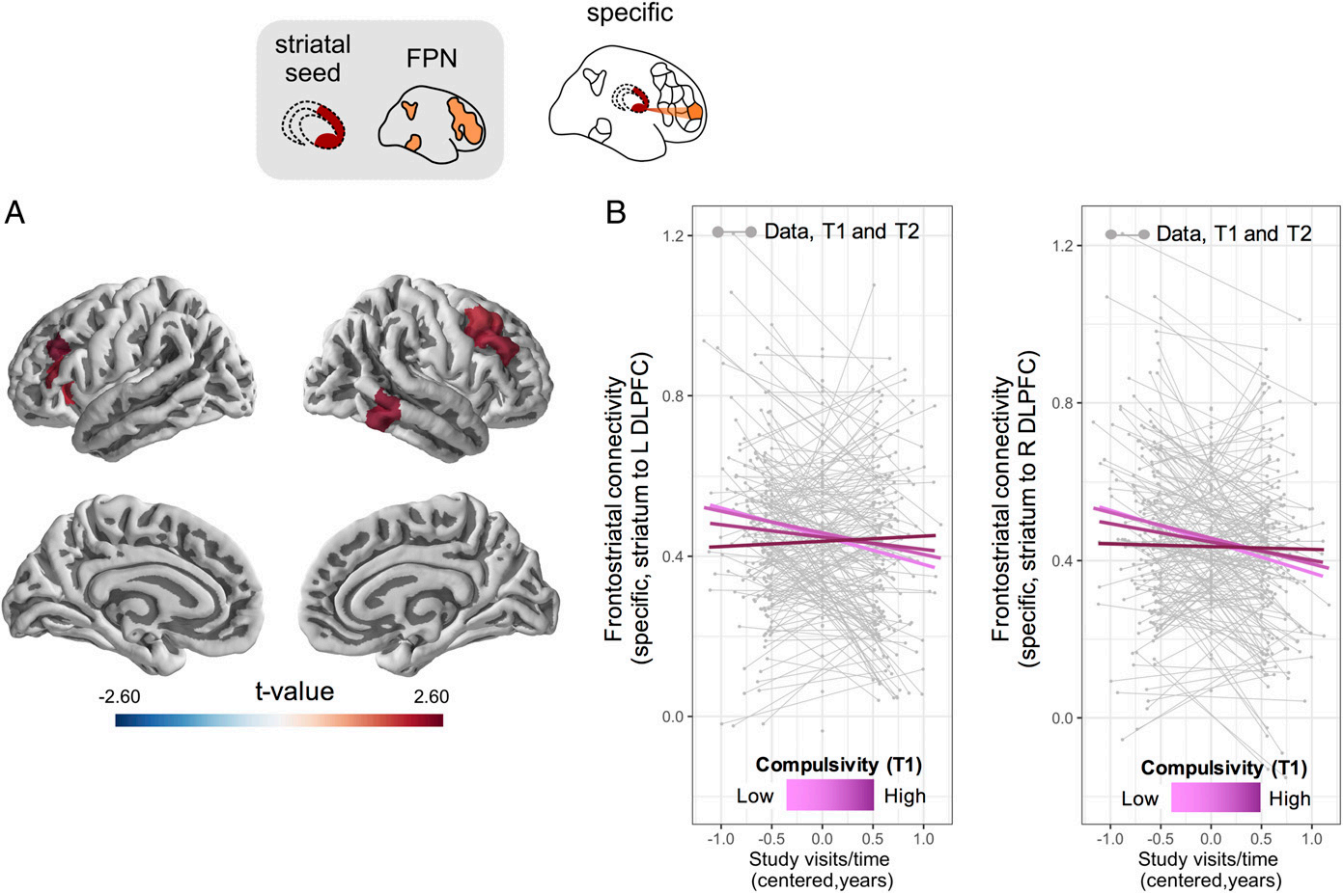
Longitudinal developmental changes in frontostriatal functional connectivity are reduced in subjects with high compulsivity. (*A*) We investigated regional specificity of the relationship between compulsivity and within-subject changes in frontostriatal connectivity. The compulsivity-related slowing in within-subject rate of change in striatal connectivity was detected mostly in regions comprising portion of the DLPFC, inferior frontal gyrus, and anterior insula. The panel shows a thresholded statistical map of regions for which an interaction between compulsivity at T1 and visits/time was observed at *P* < 0.05 (uncorrected for multiple comparisons) (see *SI Appendix*, Fig. S3 for individual panels related to each region). (*B*) Influence of compulsivity is shown specifically for the L/R DLPFC (central portion, p9-46v; L DLPFC: β = 0.008, SE = 0.003, df = 173, *t* = 2.581, *P* = 0.011; R DLPFC: β = 0.007, SE = 0.003, df = 173, *t* = 2.30, *P* = 0.023, uncorrected for multiple comparisons). These findings indicate that early in adolescence, high compulsivity traits determine slower changes in functional connectivity within frontostriatal circuits of known importance for the pathological manifestation of OCD (*n* = 230). Top schematic shows measures of functional connectivity used. Regionally specific measures of functional connectivity were estimated by computing the pair-wise correlations between the striatum and each individual cortical region in the FPN. T1, baseline; T2, follow-up.

To investigate frontostriatal connectivity development as a function of compulsivity and model-based control with greater regional specificity, we estimated pair-wise correlations between the striatum and each individual cortical region in the FPN (*Materials and Methods* and *SI Appendix*). Using an LME model of change as above, accounting for site, age, gender, and IQ, we found an effect of compulsivity at T1 on within-subject changes in connectivity for a coupling between the striatum and left/right (L/R) DLPFC (central portion, P9-46v), R DLPFC (8Av, 8C), R auditory cortex (TE1p), L anterior ventral insular area, and L inferior frontal cortex (compulsivity T1 by visits/time interaction, all *P*s < 0.05 uncorrected for multiple comparisons) (Fig. 5 and *SI Appendix*, Fig. S3). For these regions, higher compulsivity trait scores at T1 predicted a relative lack of within-subject change in frontostriatal connectivity. Comparable results were obtained when using the alternative compulsivity measure akin to that previously used to assess the relationship between compulsivity and brain markers of myelin development (37) (*Materials and Methods* and *SI Appendix*, Fig. S4). However, as these associations did not survive correction for multiple comparisons, we urge caution. Model-based control at T1 did not influence connectivity between the striatum and any of the FPN individual cortical regions.

## Discussion

Using an accelerated longitudinal design, involving a large sample of adolescents and young adults, we identified a typical trajectory of adolescent and early adult development characterized by a progressive strengthening of model-based control. We also found that the development of model-based control was more pronounced in younger participants. Interestingly, model-based control did not predict rate of change in compulsivity over time but, instead, high compulsivity related to an altered trajectory of model-based control and frontostriatal functional connectivity. Thus, higher compulsivity traits at a young age were linked to reduced development of model-based control and less pronounced within-subject change in frontostriatal connectivity.

Our longitudinal design enabled us to demonstrate a within-subject developmental increase in model-based control during the course of adolescence and young adulthood. Thus, our results extend on previous findings showing a cross-sectional effect of age on model-based control (10). An independent sensitivity analysis of a retest sample provided no evidence for a training effect as a plausible explanation. The aforementioned study (10) reported that model-based control is absent in children (i.e., 8 to 12 y), emerges in adolescence (i.e., 13 to 17 y), and strengthens further over later developmental stages (i.e., 18 to 25 y). In line with those findings, and within the narrower age range of the present sample, we found that within-subject changes were dependent on age such that an improvement in within-subject model-based control was more prominent for younger participants, and less so in those who had already reached more advanced developmental stages by the time of recruitment to the study. Therefore, in our sample it is likely that the most marked changes in model-based control had already occurred at recruitment into the study. The likelihood that older participants had already reached a plateau in model-based control at recruitment is supported also by our latent change score model, where the latter accounted for differences at baseline yet failed to identify an effect of age on rate of change in model-based control.

Consistent with the wider literature (17, 41), our study shows that higher compulsivity is associated with reduced model-based control, thereby extending this finding to a large sample of healthy adolescents drawn from the general population. More importantly, our longitudinal design and path modeling (34, 35) allowed us to capture temporal dependencies between compulsivity and model-based control, as well as to investigate separate aspects of this relationship that have heretofore been unaddressed. Thus, we identify an association between model-based control and compulsivity already at baseline, suggestive of influences operating prior to study recruitment. Additionally, after accounting for prior differences, our data show that within-subject developmental trajectories in model-based control are modulated by the presence of high compulsivity traits. Finally, the rate of model-based strengthening was less marked in those participants whose compulsivity became more severe over time. Importantly, our participants were screened for clinically diagnosed psychiatric disorder, and excluded on this basis, ensuring that compulsivity did not reflect a clinical level of impairment in the studied cohort (42). Thus, we infer that individual differences in compulsivity have relevance for maturation of model-based control, an effect which we speculate might precede pathological manifestations of OCD or other psychiatric conditions on the compulsivity spectrum.

Across multiple analyses, individual differences in compulsivity were linked not only to changes in model-based control but also to changes in frontostriatal functional connectivity. Importantly, our results were independent of the measure of compulsivity used. One limitation here is that the Leyton Obsessional Inventory-Child Version Survey (LOI) (43) has moderate reliability, and has been validated in young people alone. Reassuringly, convergent findings were obtained when we used different measures of compulsivity, suggesting our results are robust to the specific psychometric properties of individual scales (*SI Appendix*). Specifically, high compulsivity was associated with a relative lack of change in functional connectivity within a specific frontostriatal circuit, comprising the head of caudate, the putamen, and associated FPN cortical regions (39, 40). This effect was observed in the wider context of an overall within-subject longitudinal decrease in subcortical–cortical connectivity.

The developmental trajectory of resting-state functional connectivity in this sample has been previously reported (16) as showing a disruptive decrease in subcortico–cortical connectivity, as well as a more conservative pattern of increase in cortico–cortical connectivity, particularly with respect to association cortical areas. Interestingly, several areas of the striatum, such as the caudate nucleus, nucleus accumbens, pallidum, and putamen showed the greatest degree of disruptive reorganization in functional connectivity, whereby connections with cortical areas that were strong at 14 y became weaker over the course of adolescence (16). Convergent findings are evident also in other developmental fMRI studies (12–15). In particular, a longitudinal analysis from an independent study of individuals aged 8 to 29 y reported that functional connectivity between the nucleus accumbens and caudate nucleus to cortical regions weakens over the course of adolescent development (14). It has been proposed that an initial excess in connectivity is followed by a pruning process that reconfigures connectivity in the developing brain (44). Here, we add to this literature by showing that a developmental weakening in connectivity, between subcortical nuclei and frontoparietal cortical areas, is less pronounced in those individuals with higher compulsivity. It is worth noting that high compulsivity was also associated with a delayed maturational trajectory in model-based control, a relationship that might be underpinned by the very same neurodevelopmental processes.

We identified differences for the longitudinal and cross-sectional age effects on brain maturation. A detailed discussion of observed discrepancies goes beyond the scope of this paper (for more details, see ref. 45), but similar divergences have been observed in previous analyses (45, 46). In fact, the effects of within- and between-subjects component can be very different, as shown by Neuhaus and Kalbfleisch (47) and might be explained by factors such as local noise level, ground truth ratio of within- and between subject variability, the presence of sampling biases, or cohort effects for the specific measure of interest.

A secondary finding in this study was an emergence of a relationship between compulsivity and striatal connectivity with specific areas of the cortex. At an uncorrected threshold, high compulsivity related to lack of developmental change in overall connectivity of the striatum with multiple frontal regions, including the DLPFC. However, the strength of association between compulsivity and developmental change in edge-wise connectivity did not survive correction for the multiple comparisons entailed by this regionally specific approach. Nevertheless, convergent findings have been reported for this sample in relation to a different imaging biomarker, whereby high compulsivity was linked to reduced rate of within-subject change in a myelin-sensitive marker within areas of the frontal cortex corresponding to those identified here (37). More generally, frontoparietal regions are known to be implicated in OCD (26), showing perturbed connectivity (30, 33) and altered activation in response to tasks that tap goal-directed control (26, 29, 30).

We observed that individual differences in compulsivity were associated with an altered developmental trajectory of model-based control and frontostriatal connectivity. Speculatively, one possibility is that compulsivity has cascading effects. For example, in the more extreme instance of OCD, it is possible that chronic compulsive behaviors are sufficient to induce alterations in specific brain circuits (48). Similarly, anxiety or stress, core characteristics in OCD and the associated poor well-being, can influence neuromodulatory neurotransmisson, with downstream consequences on both brain connectivity and cognitive abilities, such as model-based planning (49). Alternatively, another abnormal biological process, as yet unknown, might impact on the developmental trajectory of frontostriatal networks, leading to a behavioral phenotype of high compulsivity and reduced model-based control.

Importantly, in disorders of compulsivity, such as OCD, the experimental context as much as the developmental stage might play a role in neural activations and connectivity patterns (24). Therefore, it remains to be understood how connectivity changes in relation to nonpathological and pathological levels of compulsivity. We obtained only limited evidence suggesting that frontostriatal connectivity is predictive of changes in compulsivity over time. Therefore, even if interesting, further work is needed to corroborate this finding.

Disruption within the DLPFC has been causally associated with impairment in model-based control in humans (25). More generally, studies in animals show that model-based control relies on a relative extensive network of regions, including the PFC (6). Consistent with this, we found evidence for an association between model-based control and connectivity between the striatum and the FPN. However, functional connectivity at baseline was not associated with within-subject rate of change in model-based control, nor vice versa. The findings of a within-subject decrease in compulsivity scores are consistent also with other prospectively collected data, both in adolescents (50) and adult patients (51). Even though improvement might be facilitated by therapeutic intervention, data from a large community cohort, recruited at 19 y of age and studied prospectively until 41 y old, showed a similar trajectory in healthy subjects, who reported a decrease in obsessive-compulsive scores over time (52).

In line with recent studies we identified relatively weak relationships, highlighting a need for large samples to estimate meaningful effect size. It is clear that there are only weak associations between individual differences on psychopathological dimensions and behavioral performance on neurocognitive tasks (53, 54), possibly reflecting the fact that distinct domains of cognition each make a relatively small contribution to manifest mental health disorders (55). Here, small effect sizes might also be due to relatively unstable psychometric properties of the task measures used (56). However, we point out that for this study we used a model-agnostic measure that has been shown to be superior to the one commonly derived from computational models (56).

While previous work has addressed the relationship between compulsivity and model-based control, our study is distinctive in several ways. We used an accelerated longitudinal approach in a large and population-representative sample and studied conjointly model-based behavior, individual differences in compulsivity, and functional connectivity. Furthermore, we narrowed our investigation to a specific time window within the adolescence period (14 to 24 y), which is sensitive to the emergence of many psychiatric disorders (57, 58). Finally, we made use of statistical techniques to precisely investigate the temporal dynamics of the relationship between individual differences in compulsivity and cognition, as well as state-of-the-art acquisition sequence and preprocessing methods for fMRI analysis of resting-state functional connectivity.

In conclusion, we report a large longitudinal study on the development of model-based control in adolescence and young adulthood. We show that model-based control undergoes maturational changes, which are especially pronounced in early adolescence. Critically, our results indicate also that compulsivity is related to altered adolescent model-based development and changes in frontostriatal connectivity. Thus, in an otherwise healthy sample, compulsivity is linked to atypical developmental trajectories of cognitive processes and cortico-striatal systems, known to be implicated in the clinical manifestation of OCD.

## Materials and Methods

### Design and Recruitment.

The study was approved by the Cambridge Central Research Ethics Committee (12/EE/0250), and all participants (if <16 y old, also their legal guardian) gave written informed consent. Data were obtained from a community-based longitudinal sample of healthy young people (age range 14 to 24 y old). A detailed description of the assessment procedure is provided in Kiddle et al. (42) and *SI Appendix*.

### Reinforcement Learning Task Measuring Model-Based Control.

To investigate the developmental trajectory of model-based control, we probed behavioral performance on a typical two-step reinforcement task (Fig. 1*A*) (9; see also ref. 59) (*SI Appendix*). Participants were invited to take part in a detailed in-laboratory behavioral assessment (including the reinforcement task investigated in the present study) on at least two occasions. The in-laboratory visits also included clinical interview and assessment to estimate IQ; 569 participants completed the reinforcement task investigated in the present study, at each of the two occasions (T1, baseline; T2, follow-up) (Fig. 1*B*) ∼18 mo apart (mean: 17.31 mo; median: 18 mo; SD = 3.57 mo). Following exclusion criteria based on task performance, as specified in *SI Appendix* and consistently with previous studies on this sample (56, 59), 551 participants were included as our final sample.

### Assessment of Compulsivity.

To obtain a measure of compulsivity, we analyzed psychometric questionnaires, which were administered over the course of the study. As primary measure of compulsivity, we used the short version of the LOI (43), specifically devised to measure individual differences in compulsivity in young people, with adequate sensitivity and specificity (60). More recently, the LOI has been used in a large sample of 17-y-old adolescents who were followed longitudinally over 18 mo, supporting the validity of the use of the LOI in adolescents transitioning into adulthood (61). Importantly, even if other questionnaires related to compulsivity were completed by the participants, the LOI was the only questionnaire that was available for the majority of participants at both time points. Therefore only by using this measure we were able to disentangle the reciprocal interactions between model-based control and compulsivity over time; 520 participants for whom behavioral data passed quality check, completed the LOI questionnaire shortly before the baseline (T1) in-laboratory behavioral assessment (mean: 4.50 mo; SD = 4.05 mo) and at the the follow-up (T2) in-laboratory behavioral assessment. The LOI proved to have moderate reliability as quantified by the Pearson’s correlation between T1 and T2 scores (*n* = 520, Pearson’s *r* = 0.57) (*SI Appendix*, Fig. S1*A*). Further analyses showed that the LOI captures compulsivity (*SI Appendix*).

Previous work in this sample used principal component analysis to derive an overall measure of compulsivity, to investigate the relationship between compulsivity and brain markers of myelin development (37). We performed here the same analysis by deriving a factor of compulsivity from principal component analysis on the LOI, PI-WSUR, and the revised Obsessive-Compulsive Inventory (62) available at different time points for the sample. The obtained component had a high correlation (Pearson’s *r* = 0.86) with the LOI and was used to investigate the relationship between compulsivity and functional connectivity and allow the comparison with previous work.

### Imaging Data Acquisition.

To obtain brain structural and functional measures, we conducted imaging on 306 adolescents recruited to the study (*SI Appendix*). Scanning took place at three sites, on three identical 3T whole-body MRI systems (Magnetom TIM Trio; VB17 software version; Siemens Healthcare) with standard 32-channel radio frequency (RF) receive head coil and RF body coil for transmission. Resting-state fMRI data were acquired using a multiecho echoplanar imaging (ME-EPI) sequence with online reconstruction (63): 263 volumes; repetition time = 2.42 s; Generalized Autocalibrating Partial Parallel Acquisition (GRAPPA) with acceleration = 2; matrix size = 64 × 64 × 34; field-of-view = 240 × 240 mm; in-plane resolution = 3.75 × 3.75 mm; slice thickness = 3.75 mm with 10% gap, 34 oblique slices; bandwidth = 2,368 Hz per pixel; echo time = 13, 30.55, 48.1 ms. Preprocessing of imaging data has been previously described for the sample included in this study in Váša et al. (16) and *SI Appendix*. For the present study, we retained 209 scans for T1, corresponding to participants whom measures of model-based control and compulsivity were also available for the corresponding time point; similarly, 199 scans were retained for T2. Within the final sample, 178 participants were scanned twice and 52 participants were scanned once (*n* = 230 subjects).

### Parcellation and Functional Connectivity Estimation.

Preprocessed images were parcellated using a recent multimodal template based on data from the Human Connectome Project (HCP) (64), yielding 360 bilaterally symmetric cortical regions. As the aim of this study was to focus on connectivity within frontostriatal circuits, a functionally principled parcellation of the striatum was used leveraging the seven-network functional striatal atlas of Choi et al. (39) http://surfer.nmr.mgh.harvard.edu/fswiki/StriatumParcellation_Choi2012. This parcellation results from prior resting-state functional connectivity analyses and characterizes the human striatum based on resting-state functional connectivity to the cerebral cortex (39). The seven striatal regions correspond to zones linked to separate cortical (motor, ventral attention, frontoparietal, default, limbic, dorsal attention, and visual) networks from Yeo et al. (40). Therefore, for each participant, we parcellated brain data into 367 regions. Regional mean time series were estimated by averaging the fMRI time series over all voxels in each parcel. Some regions (particularly near the frontal and temporal poles) were excluded because of low regional mean signal, defined by a low *z*-score of mean signal intensity in at least one subject (*z* < −1.96); this resulted in the exclusion of 30 cortical regions from the HCP parcellation.

We a priori selected the central lateral zone of the striatum as the seed for our connectivity analysis since this striatal subregion has been shown to be preferentially coupled to the FPN (39). At the cortical level, we first mapped the HCP cortical parcellation to each of the seven cortical networks previously defined by independent component analysis of resting-state fMRI (40). Then, we selected cortical regions belonging to the FPN (see ref. 40 for mapping procedure) (see *SI Appendix*, Fig. S2 and Table S11 for a complete list of the 42 cortical regions included within the FPN). Functional connectivity matrices were estimated by Pearson’s correlation between each pair of cortical and striatal regional mean time series.

Frontostriatal connectivity was estimated at multiple spatial scales. First, we estimated an overall striatal connectivity strength, defined as the average functional connectivity of the striatal seed region to all cortical regions included in the FPN. Second, we estimated specific striatal connectivity strength as the pair-wise functional connectivity of the striatal seed region to each individual cortical region included in the FPN.

### Analysis of the Reinforcement Learning Task, Measuring Model-Based Behavior.

Logistic regression analysis of this task has been widely applied (9, 65) and we used it here to analyze choice behavior. Logistic regression analyses were conducted using the lme4 package in the R software environment [R Development Core Team, 2016, v3.1.1 (66)] (*SI Appendix*). Accordingly, we specified a mixed-effects logistic regression to explain the first-stage choice on each trial *t* (coded as stay vs. switch) using binary predictors, indicating if a reward was received at *t* − 1 and the transition type (common or rare) that had produced it. To obtain a per subject measure of model-based control, we used the estimated coefficients of the reward by transition interaction. Similarly, to obtain a per subject measure of model-free strategy, we used the estimated coefficient of the main effect of reward. These measures, estimated separately for T1 and T2, were used for the main longitudinal analyses, which included appropriate covariates as explained below.

### Developmental Changes in Model-Based Control and Compulsivity.

To investigate developmental changes of model-based control, we used the estimated coefficients of the reward by transition interaction at each time point in the context of LME modeling. We followed analysis recommendations (36) successfully adopted in recent studies from this sample (37, 38) (see also *SI Appendix*).

Briefly, taking advantage of the accelerated longitudinal design, we were able to study separately (in one joint model): 1) How model-based control changed within subjects (from T1 to T2), and 2) how model-based control varied between subjects as a function of mean age of the participant and 3) their interaction. This latter factor indicates how changes over time vary according to the mean age of the subject, independently of other covariates included in the model (see *SI Appendix* for more details). The model included gender and IQ, which have been previously reported to covary with goal-directed behavior (67–69). As IQ scores were highly correlated across sessions (*r* = 0.77, *P* < 0.001), the two measurements were collapsed in an average value per participant, then *z*-scored and entered in the regression model as a covariate. The model also included all of the first-order interactions among all of the variables included in the model. This analysis included 541 participants (i.e., those participants who had model-based and IQ measures available at both time points). An equivalent model was separately implemented to investigate developmental changes in compulsivity and a possible modulation of individual differences in compulsivity dependent on mean age of the subject (*SI Appendix*, Table S4). This analysis demonstrated within-subject changes in compulsivity, which generally decreased over time (β = −1.183, SE = 0.162, df = 516, *t* = −7.30; *P* < 0.001). The within-subject rate of improvement in compulsivity was not conditioned by mean age of the subject (β = 0.064, SE = 0.040, df = 516, *t* = 1.62; *P* = 0.107).

To determine whether the observed longitudinal differences were predominantly due to retest effects (i.e., familiarity with the task, practice effect) or development, we considered data from a subsample of participants who completed the reinforcement learning at an additional time point shortly after (∼6 mo) the baseline measurement. This T1R allowed us to isolate a possible training effect, indexed by short-term changes, from developmental changes, indexed by long-term changes. We performed a logistic regression that included trial-by-trial data of all participants from the three time points (T1, baseline; T1R, retest; T2, follow-up) (i.e., syntax of R as follows: Stay ∼ Reward * Transition * (IQ_*zscore*_ *+ Age*_*zscore*_ + Gender + Time point) + (Reward * Transition * Time point + 1 | Subject). We hypothesized that developmental changes in model-based control should be expected only when 18 mo had elapsed between measurements and not for measurements separated only by 6 mo. Therefore, a reward-by-transition type by session interaction was hypothesized for the follow-up (T2) but not the retest assessment (T1R). A finding consistent with our hypothesis would be suggestive that model-based control improves as function of developmental maturation and not simply as a function of repetition of the task per se. Fifty-three participants were included in this analyses, having completed the reinforcement learning task at T1, T2, and at the retest time point (*SI Appendix*, Table S5).

### Model-Based Control and Compulsivity.

Previous studies have shown an association between increased compulsivity and reduced model-based control in putatively healthy adults (70) and in adults and adolescents affected by OCD (17, 41) or other compulsivity disorders (17). We tested whether this association could be identified in our sample with a logistic regression model, which examined if participants’ behavior on the reinforcement learning task was influenced by individual differences in compulsivity. This logistic regression, which included data from T1 and T2, had compulsivity scores (*z*-scored), IQ (*z*-scored), age (*z*-scored), and gender, with all two-way and three-way interactions as fixed effects. Within-subject factors were allowed to vary across participants by specifying the per participant random adjustment to the fixed intercept (random intercept) and the per participant adjustment to previous reward, transition-type, and their interaction (random slopes). The main measure of interest was the three-way interaction between reward, transition type, and compulsivity. The model was specified in R as follows: Stay ∼ Reward * Transition * (IQ_*zscore*_ *+ Age*_*zscore*_ *+ LOI*_*zscore*_ + Gender) + (Reward * Transition + 1 | Subject) (*SI Appendix*, Table S6).

### Longitudinal Relationships between Model-Based Control and Compulsivity.

Having identified developmental changes in model-based control, and an association between model-based and compulsivity, we wanted to obtain a more detailed understanding of their reciprocal influences over time. We asked whether individual differences in compulsivity could predict the maturational trajectory of model-based control and, vice versa, whether model-based control could predict the course of compulsivity over time. Such a notion is known as cross-domain coupling and can be captured by bivariate latent change score models (34). These allowed us to evaluate to what extent longitudinal changes in one domain (e.g., model-based control) are guided by baseline scores in the other domain (e.g., compulsivity). Importantly, this association is adjusted for initial, baseline dependency.

Here, model-based control was indexed by the estimated coefficients of the reward by transition interaction obtained previously for each participant at T1 and T2; compulsivity was indexed by the LOI scores at T1 and T2 (Fig. 3 and *SI Appendix*, Table S7). Given that gender and IQ, as well as age, showed relationship with model-based control and that inclusion or exclusions of covariates can influence the relations between individual variables (71), a separate model included age, gender, and IQ, which were regressed both on the observed variables at T1 and on the latent change variables of both model-based and compulsivity. We allowed for residual covariance between demographic variables (*SI Appendix*, Table S8). We verified specificity of our findings by testing a separate model, which included the estimates of the main effect of reward, putatively indexing the model-free component, instead of the reward-by-transition interaction term. To test for convergent validity of our results, we used the PI-WSUR instead of LOI to index compulsivity at T1 and T2.

The models were estimated in the *lavaan* software package (72) in R (R Development Core Team, 2016) (66) using full information maximum likelihood (‘mlr’ implemented in *lavaan*) with robust SE to account for nonnormality. There were no missing data. We assessed overall model fit via the χ^2^ test, the RMSEA (acceptable fit: < 0.08, good fit: < 0.05), the CFI (acceptable fit: 0.95 to 0.97, good fit: > 0.97), and the SRMR (acceptable fit: 0.05 to 0.10, good fit: < 0.05) (73).

### Longitudinal Relationships between Model-Based Control, Compulsivity, and Frontostriatal Connectivity.

To investigate reciprocal influences between compulsivity, model-based, and functional connectivity, we extended our bivariate latent change score model by adding the overall striatal strength at T1 and T2. Site was regressed on connectivity measures (Fig. 4 and *SI Appendix*, Table S9) to account for differences in scanning sites. Similar to the previous implementation to investigate the relationship between model-based control and compulsivity, a separate model included not only site, but also age, gender, and IQ, which were regressed both on the observed variables at T1 and on the latent change variables of model-based, compulsivity, and functional connectivity. We allowed for residual covariance between demographic variables (*SI Appendix*, Table S10).

To validate our findings, we also used an alternative analytical approach leveraging an LME model akin to the one used previously. Here, we tested how changes in the overall striatal connectivity strength were determined by different factors. We included age and visits/time (computed as explained in *SI Appendix*,) to distinguish between- and within-subject components of change in overall striatal connectivity strength dependent on age. In addition, this model, as above, included site, gender, and IQ as covariates. Model-based control and compulsivity scores at T1 as well as their interaction with visits/time were also included. These latter interactions (compulsivity at T1 by visits/time and model-based control at T1 by visits/time) tested how changes of overall connectivity strength over time were modulated by initial model-based control and compulsivity, independently of other covariates included in the model. This same analytical approach was implemented to investigate the role of compulsivity on regionally specific striatal connectivity to each of the FPN cortical regions.

As a control analysis, to assess selectivity of our findings, we applied the same analytical approach to investigate if compulsivity affected connectivity between this same striatal region and cortical regions of another network, namely the motor one as defined in Yeo et al. (40). Finally, to test robustness of our findings, we fitted linear models of change of both overall and specific striatal strength maps as a function of an alternative compulsivity score, obtained by using principal component analysis as described above and in Ziegler et al. (37).

## Supplementary Material

Supplementary File

## Data Availability

Data have been deposited on Open Science Framework (https://osf.io/vm62u/).
