# Supplementary material for: Compulsivity is linked to reduced adolescent development of goal-directed control and frontostriatal functional connectivity
Source: Proc Natl Acad Sci U S A. 2020 Sep 28;117(41):25911–22. doi: 10.1073/pnas.1922273117 (PMC7568330; doi:10.1073/pnas.1922273117)
Supplement: Supplementary File [file pnas.1922273117.sapp.pdf]

Supplementary Information for

Compulsivity is linked to reduced adolescent development of goal-directed control and fronto-striatal functional connectivity

Matilde M. Vaghi, Michael Moutoussis, František Váša, Rogier A. Kievit, Tobias U. Hauser, Petra E. Vértes, Nitzan Shahar, Rafael Romero-Garcia, Manfred G. Kitzbichler, Edward T. Bullmore, NSPN Consortium, Raymond J. Dolan

Matilde M. Vaghi
matilde.vaghi@gmail.com

Contents

Compulsivity is linked to reduced adolescent development of goal-directed control and fronto-striatal functional connectivity 1

Participant's recruitment. 3

Reinforcement learning task measuring model-based control. 3

Exclusion criteria for the reinforcement learning task. 4

Assessment of compulsivity. 4

Imaging data acquisition and pre-processing. 5

Exclusion criteria for the imaging data. 6

Analysis of the reinforcement learning task. 6

Longitudinal statistical analyses. 7

Effects of compulsivity, assessed with a secondary measure, on development of model-based control. 8

Effects of compulsivity, assessed with a secondary measure, on development of fronto-striatal connectivity. 9

Table S1. Results from Basic Logistic Regression Model at Baseline and Followup used to extract per subject estimates of model-based and model-free learning. 10

Table S2. Results from linear mixed modeling to investigate development of model-based control. 10

Table S3. Results from linear mixed modeling to investigate development of model-free learning 11

Table S4. Results from linear mixed modeling to investigate development of compulsivity as measured by the Leyton Obsessional Inventory 11

Table S5. Results from the logistic regression to investigate model-based improvement when the task was completed shortly after the first assessment (retest) and after prolonged time (Follow-up) when developmental changes are expected 12

Table S6. Results from the logistic regression to investigate association between model-based and compulsivity 13

Table S7. Bivariate latent change score model between compulsivity and model-based control 14

Table S8. Bivariate latent change score model between compulsivity and model-based control (age, gender and IQ were regressed on observed and latent variables) 15

Table S9. Extended latent change score model between compulsivity, model-based control, and fronto-striatal connectivity 17

Table S10. Extended latent change score model between compulsivity, model-based control, and fronto-striatal connectivity (age, gender and IQ were regressed on observed and latent variables) 19

Table S11. List of cortical regions selected based on frontoparietal mask from Yeo et al., 2011 22

Table S12. Neuroscience in Psychiatry Network (NSPN) consortium author list 23

Figure S1. Stability and convergent validity of compulsivity questionnaire 24

Figure S2. Display of cortical brain regions included within cortical frontoparietal mask 24

Figure S3. Regional effects of compulsivity on development of fronto-striatal connectivity 25

Figure S4. Overall and regional effects of compulsivity using a composite compulsivity measure 26

Figure S5. Effects of head motion on variables of interest 27

Supplementary References 28

Participants' recruitment. In total, 2,406 healthy young people (age range 14-24 years old) were recruited in an accelerated longitudinal study from schools, colleges, National Health Service (NHS) primary care services, and via direct advertisement in north London and Cambridgeshire as part of the NSPN study (1). Subjects were stratified into contiguous age-related strata: 14-15 years inclusive, 16-17 years, 18-19 years, 20-21 years, and 22-24 years, with roughly equal number of subjects per bin, and equal gender and ethnicity. All participants satisfied the following eligibility criteria: aged between 14 and 24 years inclusive; able to understand written and spoken English; willing and able to give informed consent for recruitment into the study and consent to be re-contacted directly for possible participation in future studies within the Consortium. They were excluded if they were currently participating, or had recently (within the last 12 months) participated, in a clinical trial. Participants were excluded if they were currently being treated for a psychiatric disorder or for drug or alcohol dependence; had a current or past history of neurological disorder or trauma; or had a learning disability. Participants were invited to take part in a detailed in-lab behavioral assessment (including the reinforcement task investigated in the present study) on at least two occasions. Finally, for some of the subjects who participated to the in-lab visits, an imaging session was conducted to obtain brain structural and functional measures (see Imaging Section). Recruitment of the imaging cohort was conducted by inviting participants from each age-sex-stratum in equal numbers. They could participate if they had no safety contraindication on the MRI scanning checklist. Further details about recruitment, participant's consent, and ethical approval can be found at (36).

Reinforcement learning task measuring model-based control. The design of the behavioral probe is analogous to previous implementations of the task (2) and described in details for this sample in (3). On each trial the participant was presented with a choice between two fractals (first stage) (2000 ms choice window). Selecting one of the fractals led to another two fractals choice (second stage), resulting in participants being probabilistically rewarded with a reward (gold coin). Probability of being rewarded at the second stage, was fixed and drifted slowly and independently over time (never being less than 0.2 or greater than 0.8). Critically however, each fractal in the first stage commonly (70%) or uncommonly (30%) led to a particular second stage. Such experimental manipulation (i.e., transition type) allowed us to distinguish different learning strategies. A purely habitual, "model-free", learner would only base the behavioral strategy on whether or not a reward was obtained in the immediately prior trial, regardless of whether the transition was rare or common. In contrast a goal-directed, "model-based", learner would also take into account the environmental contingency considering not only whether a reward was obtained the last time they performed the action but also the transition structure of the task. The main statistical analyses we employed

capitalized on this reasoning by using logistic regression to identify whether selection of the fractals in the first stage from one trial to the next was informed only by the outcome of the prior trial (rewarded, unrewarded) or also by the transition type (common, uncommon). The reinforcement learning task included 121 trials at baseline (T1) and at the retest assessment 6 months later (T1R) due to initial time constraints. For the follow-up (T2) assessment, the number of trials was increased to 201 to match previous studies (2). Participants were instructed to win as much reward (play pounds) as possible, and were told they would receive a payment bonus based on task performance. Before completing the two-step reinforcement learning task, participants were always administered instructions and a comprehension test associated with it.

Exclusion criteria for the reinforcement learning task. Subjects were excluded if they missed more than 10% of trials (N = 3) either at T1 or T2, if they responded on the same key on more than 95% of trials on which they registered a response (N = 2) or had implausibly fast reaction times, i.e., below 150 ms on more than 20% of the trials (N = 13). This resulted in the inclusion of 551 participants in our full analysis (280 females; T1: mean age= 18.44 years, range = 14.10-24.98, sd = 2.97; T2: mean age= 20.32 years, range = 15.11-26.48, sd = 2.98). However, results presented in this paper are not substantially changed by the inclusion or exclusion of these subjects in the analyses. For the two-stage task data, the first trial in each block as well as trials with implausibly fast response times (below 150ms) were omitted from the analysis (less than 1% of the overall trials). 53 of these participants also completed the same reinforcement task at an intermediate time point (T1R), approximately 6 months after the first lab visit at T1. Additionally, following (4), we identified those subjects (T1, N = 20; T2, N = 13) who repeated previously rewarded second-stage responses at a rate lower than 50%. Inclusion or exclusion of these subjects did not impact the main results.

Assessment of compulsivity. We investigated LOI concurrent validity by testing its relationship with a separate instrument, the Padua Inventory Washington State University Revision (PI-WSUR) (5), also designed to measure compulsivity. The latter was included in the battery but it was available for a significant lower number of participants (only N=287 subjects for whom model-based scores were available at T1 and T2, also had PI-WSUR available for both assessments). At both time points (**Figure S1 B, C**), there was a high correlation between compulsivity measured by these two questionnaires (T1, N = 277, Pearson's r = 0.58; T2, N = 519, Pearson's r = 0.70). We also tested the concurrent validity of the LOI by using a composite score of compulsivity. We performed principal component analysis on all available items of the available questionnaires that probed individual differences in compulsivity (i.e., PI-WSUR and the revised Obsessive-Compulsive Inventory

(6)). This score was constructed independently of the LOI yet was highly correlated with it (mean values across assessments, Pearson's r = 0.76), concurrently validating the LOI as an appropriate measure of compulsivity in this sample. Altogether, these findings support the notion that the LOI captures compulsivity.

Imaging data acquisition and pre-processing. Pre-processing of imaging data has been previously described for this sample (7) and it is summarised here. Following processing of individual structural scans using FreeSurfer v5.3.0 (including skull-stripping, segmentation of cortical grey and white matter and reconstruction of the cortical surface and grey-white matter boundary) (8), all scans were stringently quality controlled by re-running the reconstruction algorithm after the addition of control points and white matter edits (as described previously (9, 10)). The pre-processing of functional data with ME-ICA analysis was performed using AFNI (11). Volumes acquired during steady-state equilibration (15 s) were omitted. The data of the middle TE were used to compute parameters of motion correction and anatomical-functional co-registration.The first volume after equilibration was used as the base EPI image. Matrices for deobliquing and six-parameter rigid body motion correction were computed. Then, 12-parameter affine anatomical-functional co-registration was computed using the LPC cost functional (12), using the EPI base image as the LPC weight mask. Matrices for deobliquing, motion correction, and anatomical-functional co-registration were combined into a single alignment matrix using the concatenation approach from the AFNI tool align_epi_anat.py. The dataset of each TE was then slice-time corrected and spatially aligned through application of the alignment matrix. Co-registration of structural and functional scans for both time points was visually assessed.

We used ME- ICA (13, 14) for pre-processing of functional scans to identify the sources of variance in the fMRI time series that scaled linearly with TE and could therefore be confidently regarded as BOLD signal. Other sources of fMRI variance, such as head movement, which were not BOLD-related and therefore did not scale with TE, were identified by ME-ICA and discarded. The retained independent components, representing BOLD contrast, were optimally recomposed to generate a broadband denoised fMRI time series at each voxel. This was bandpass filtered by the discrete wavelet transform (Daubechies 4 wavelet), resulting in a BOLD signal oscillating in the frequency range 0.025-0.111 Hz (wavelet scales 2 and 3). During pre-processing, realignment of scans was used to estimate 6 motion parameters for each participant (3 translation parameters and 3 rotation parameters). Subsequently, these were used to calculate an overall estimate of motion - the framewise displacement (FD; defined as the sum of the absolute derivatives of the six motion parameters, following conversion of rotational parameters to distances by computing the arc length displacement on

the surface of a sphere with radius 50 mm as in (15, 16)). Mean FD was used as a measure of head movement in each scan session. Finally, as previously described (7), confounding effects of head movement on connectivity after ME-ICA pre-processing were corrected by regressing functional connectivity on mean FD (17, 18). Accordingly, there was no distance dependence on the relationship between residual functional connectivity (mean-FD corrected) and FD (7). Residual estimates of overall striatal connectivity (mean-FD corrected) were not correlated with head motion; and there was no significant relationship between FD and any of the measures of interest (i.e., age, model-based control, and compulsivity) (Supplementary Figure S5). Thus we used this movement correction pipeline of ME-ICA followed by FD regression as the basis for further analysis of functional connectivity.

Exclusion criteria for the imaging data. Of the total sample, 36 scans were excluded. 17 scans were excluded due to high in-scanner motion (FD > 0.3 mm or maximum FD > 1.3 mm), 9 due to coregistration errors, 7 due to a lack of non-convergence of the multi-echo independent component analysis preprocessing algorithm (Supplementary Material), 2 due to parcellation errors, and 1 due to extensive signal dropout.
Following quality control and participant exclusion, the final evaluable dataset included 298 adolescents (151 females), scanned a total of 492 times as in (7). Of these, 281 scans were acquired at baseline (T1) and 211 were acquired approximately a year later at follow-up (T2). For the present study, we retained 209 scans for T1, corresponding to participants whom measures of model-based control and compulsivity were also available for the corresponding time point; similarly, 199 scans were retained for T2. Within the final sample, 178 participants were scanned twice and 52 participants were scanned once (N=230 subjects). Imaging data were collected within maximum 2 months (mean: 0.11 months; median: 0 months; SD = 0.36 months) or 5 months (mean: 0.28 months; median: 0 months; SD = 0.83 months) from the T1 or T2 behavioral assessments, respectively.

Analysis of the reinforcement learning task. Logistic regression analysis has been widely applied for the analysis of the reinforcement learning task used in this study. Logistic regression analyses were conducted using generalized linear mixed effects modelling with the lme4 package in R software environment (R Development Core Team, 2016, version, 3.1.1). Firstly, we conducted a basic logistic regression separately for T1 and T2 to test if participants' choice behavior (coded as: switch= 0; stay = 1, relative to the previous choice) was influenced by reward (coded as: rewarded = 1; unrewarded = -1), transition (coded as: common = 1; rare = -1), and their interaction, on the preceding trial. A model-free learning strategy would predict a main effect of reward on stay probability. This is because model-free choices simply rely on the outcome obtained, regardless of the structure of the environment (i.e., whether the trial is

rewarded following a common or rare transition). In contrast, a model-based control strategy would predict an interaction between reward and transition as a rare transition reverses the effect of a subsequent outcomes (**Figure 1A**). Within-subject factors (the intercept, main effects of reward and transition, and their interaction) were modelled as random effects, i.e. allowed to vary across subjects. We used Bound Optimization by Quadratic Approximation (bobyqa) with 1e5 functional evaluations. For each time point, the model was specified in the syntax of R as follows: *Stay ~ Reward * Transition + (Reward * Transition + 1 | Subject).* Individual dependent variables for following analysis were defined based on these random effects.

Longitudinal statistical analyses. To investigate longitudinal changes in model-based and compulsivity as well as to study how longitudinal changes of fronto-striatal connectivity were affected by individual differences in model-based control and compulsivity we used linear mixed models. Importantly, within these models, we decomposed age into the two separable components: a between-subject component and a within-subject component as explained in further detail in (19). These are generally conflated in the context of cross-sectional studies but it is possible to decompose them in a longitudinal observational design (20). The between-subject component (further referred to as *age_mean*) is purely cross-sectional and can be obtained by considering only each participants' mean age across all their measurements centred based on the mean of the sample (i.e., *age_mean* = age_i- $mean\ age\ sample$). The within-subject component (further referred to as *visits/time*) is purely longitudinal and considers the effect of study visit on each participant and follows by subtracting the mean age of a participant from its age covariate (i.e. effect of *visits/time* = $age_{it} - age_i$).
Thus, here we split the age covariate into its between and within-subject components and systematically included both in our linear mixed models. For the analysis on longitudinal changes in model-based control (see section Developmental changes in model-based control and compulsivity and **Table S2**) and the corresponding one on compulsivity (reported in Developmental changes in model-based control and compulsivity and **Table S4**) the effects of interest were the study *visits/time* and *age_mean* as well the interaction between *visits/time and age_mean* to test for different rates of change (over study) in subjects with higher or lower age. These models also included gender and mean subject IQ (Z-scored) and the interactions between visits/time and each of these covariates to test if changes (over study) were dependent on being male/female or on mean IQ as these two variables have been shown to be relevant for model-based and for expression of OCD symptoms.

To investigate if functional changes in fronto-striatal connectivity were guided by compulsivity and/or model-based control (see section High Compulsivity Is Associated with Reduced

Developmental Changes in Striatal Connectivity**)** mixed models were also used. The effects of interest were the study *visits/time* and *age_mean* as well the main effects of compulsivity, model-based and their interaction with study visits/time (i.e., *visits/time by compulsivity* interaction; *visits/time by model-based* interaction). The visits/time interactions would allow testing for different rates of change in functional connectivity for subjects with higher or lower initial model-based and / or compulsivity. Visits/time refers to the individually centered study time as introduced above (and separated from age_mean differences). Notably the inclusion of the main effect of model-based control and compulsivity renders the observed *visits/time by model-based* interaction *and visits/time by compulsivity* interaction effects independent from existing differences across individuals at baseline (statistically decoupling past differences form ongoing change). These models also included the gender, subject IQ, and site.

Effects of compulsivity, assessed with a secondary measure, on development of model-based control. To test generalizability of our findings on the relationship between compulsivity and within-subject changes in model-based control, we implemented the bivariate latent change score model by using an alternative compulsivity measure. Namely, scores of compulsivity were obtained from the Padua Inventory Washington State University Revision (PI-WSUR) (5). The latter was included in the battery but it was available for a significant lower number of participants (Materials and Methods). Results largely replicated those obtained when using LOI and reported in the main text. There was a significant negative correlation between the model-based control and compulsivity, here measured with the PI-WSUR, at T1 (Z-value= -2.451, P = 0.014, standardized estimate = -0.12). Also consistent with the model reported in the main text, subjects differed in the rate of change in compulsivity (Z-value = 7.807, $P < 0.001$, standardized estimate = 0.85) and model-based control (Z-value = 12.389, $P < 0.001$, standardized estimate = 0.63) as indicated by the significance associated with the respective variances in the rate of change. The association between compulsivity and rate of change in model-based control was in the same direction and of slightly larger (standardized) magnitude as the one reported in the main text using LOI, albeit not nominally significant, likely due to the difference in sample size (Z-value = -1.841, P = 0.066, standardized estimate = -0.086). As for the previous analysis, a path testing whether initial model-based control predicted compulsivity changes over time was not significant (Z-value = -1.616, P = 0.106, standardized estimate= -0.075). There was not a residual correlation between the rates of change (Z-value = -1.254, P = 0.210, standardized estimate = -0.075). Model fit was good for this model (N=287, χ_2 = 0.098, df = 1, P = 0.755; RMSEA = 0.000 [0.000, 0.107], SRMR = 0.003, CFI = 1.000, Yuan–Bentler scaling correction factor = 1.004).

Effects of compulsivity, assessed with a secondary measure, on development of fronto-striatal connectivity. A parallel work on this sample (19) has investigated the relationship between compulsivity and a myelin sensitive imaging biomarker. An alternative measure of compulsivity was derived applying principal component analysis on all available compulsivity measures on this sample (19). To investigate the relationship between compulsivity and our imaging functional marker, we followed a similar approach (Materials and Methods) and computed a measure of compulsivity derived from principal component analysis on all available compulsivity measures on this sample. As reported in the main text, even by using this secondary compulsivity measure, we found that compulsivity affected the rate of within-subject change of overall striatal connectivity strength (β= 0.161, SE = 0.007, df = 178, t = 2.186, P = 0.0301). Similarly, when using this secondary measure of compulsivity, as reported in the main text, slowing in within-subject rate of change in specific striatal connectivity was detected mostly in regions comprising portions of the dorsolateral prefrontal cortex, inferior frontal gyrus, and the anterior insula (**Figure S4**).

Table S1. Results from Basic Logistic Regression Model at Baseline and Followup used to extract per subject estimates of model-based and model-free learning.

	Baseline (N = 551)					Follow-up (N = 551)				
**Coefficient**	**β**	**SE**	**Z-value**	***P***		**β**	**SE**	**Z-value**	***P***	
(Intercept)	1.20	0.05	24.83	**<.001**	***	1.34	0.04	29.90	**<.001**	***
Reward	0.27	0.02	14.85	**<.001**	***	0.30	0.02	19.89	**<.001**	***
Transition	0.04	0.01	2.90	**0.004**	**	0.07	0.01	6.88	**<.001**	***
Reward x Transition	0.19	0.02	11.34	**<.001**	***	0.23	0.01	15.37	**<.001**	***

*p<.05, **p<.01, *** p <.001, SE = Standard Error

Table S2. Results from linear mixed modeling to investigate development of model-based control.

**Coefficient**	**β**	**SE**	**DF**	**t**	***P***	
Intercept	0.191	0.010	537	19.07	**<.001**	***
Visits/time	0.024	0.009	537	2.63	**0.009**	**
Gender	0.035	0.014	537	2.46	**0.014**	**
IQ (Z-score)	0.052	0.008	537	6.75	**<.001**	***
Age_mean	0.006	0.002	537	2.28	**0.023**	*
Visits/time x Gender	-0.002	0.013	537	-0.15	0.877	
Visits/time x IQ (Z-score)	0.010	0.007	537	1.37	0.173	
Visits/time x age_mean	-0.005	0.002	537	-2.26	**0.024**	*

N=541. *p<.05, **p<.01, *** p <.001, SE = Standard Error, DF= Degrees of Freedom. See supplementary methods for notation on *visits/time* and *age_mean*

Table S3. Results from linear mixed modeling to investigate development of model-free learning

Coefficient	β	SE	DF	t	*P*	
Intercept	0.280	0.011	537	25.66	**<.001**	***
Visits/time	0.020	0.010	537	1.95	0.051	
Gender	-0.007	0.016	537	-0.46	0.647	
IQ (Z-score)	0.028	0.008	537	3.33	0.001	**
Age_mean	0.011	0.003	537	4.07	**<.001**	***
Visits/time x Gender	0.010	0.015	537	0.65	0.515	
Visits/time x IQ (Z-score)	-0.014	0.008	537	-1.76	0.078	
Visits/time x age_mean	-0.003	0.002	537	-1.19	0.235	

N=541, *p<.05, **p<.01, *** p <.001, SE = Standard Error, DF= Degrees of Freedom. See supplementary methods for notation on *visits/time* and *age_mean*

Table S4. Results from linear mixed modeling to investigate development of compulsivity as measured by the Leyton Obsessional Inventory

Coefficient	β	SE	DF	t	*P*	
Intercept	3.857	0.229	516	16.85	**<.001**	***
Visits/time	-1.183	0.162	516	-7.30	**<.001**	***
Gender	-0.297	0.329	516	-0.90	0.367	
IQ (Z-score)	-0.787	0.177	516	-4.45	**<.001**	***
Age_mean	0.101	0.056	516	1.81	0.071	
Visits/time x Gender	0.222	0.235	516	0.94	0.346	
Visits/time x IQ (Z-score)	0.085	0.130	516	0.66	0.512	
Visits/time x age_mean	0.064	0.040	516	1.62	0.107	

N =520, *p<.05, **p<.01, *** p <.001, SE = Standard Error, DF= Degrees of Freedom. See supplementary methods for notation on *visits/time* and *age_mean*

Table S5. Results from the logistic regression to investigate model-based improvement when the task was completed shortly after the first assessment (retest) and after prolonged time (follow-up) when developmental changes are expected

Coefficient	β	SE	Z-value	P	
(Intercept)	1.143	0.176	6.49	**<.001**	***
Reward	0.205	0.045	4.59	**<.001**	***
Transition	0.003	0.044	0.08	0.938	
Session (Retest)	0.092	0.118	0.78	0.433	
Session (Follow-up)	0.384	0.139	2.76	**0.006**	**
IQ	0.223	0.098	2.26	**0.024**	*
Age	0.239	0.109	2.29	**0.022**	*
Gender	-0.018	0.194	-0.09	0.925	
Reward x Transition	0.103	0.058	1.77	0.077	.
Reward x Session (Retest)	0.109	0.062	1.77	0.077	.
Reward x Session (Follow-up)	0.121	0.057	2.11	**0.035**	*
Reward x IQ	-0.013	0.026	-0.50	0.618	
Reward x Age	0.083	0.030	2.79	**0.005**	**
Reward x Gender	-0.080	0.051	-1.56	0.119	
Transition x Session (Retest)	0.011	0.056	0.20	0.840	
Transition x Session (Follow-up)	0.092	0.053	1.73	0.084	.
Transition x IQ	0.007	0.022	0.30	0.761	
Transition x Age	0.006	0.025	0.26	0.797	
Transition x Gender	0.060	0.044	1.39	0.166	
Reward x Transition x Session (Retest)	0.006	0.071	0.09	0.933	
Reward x Transition x Session (Follow-up)	0.127	0.062	2.06	**0.039**	*
Reward x Transition x IQ	0.063	0.031	2.04	**0.041**	*
Reward x Transition x Age	0.026	0.035	0.75	0.455	
Reward x Transition x Gender	0.118	0.060	1.96	**0.050**	*

N=53, *p<.05, **p<.01, *** p <.001, SE = Standard Error

Table S6. Results from the logistic regression to investigate association between model-based and compulsivity

	**β**	**SE**	**Z-value**	***P***	
Intercept)	1.186	0.051	23.266	**<.001**	***
Reward	0.262	0.017	15.47	**<.001**	***
Transition	0.037	0.012	3.23	**0.001**	**
IQ (Z-scored)	-0.047	0.017	-2.77	**0.006**	**
Age (Z-scored)	0.295	0.035	8.36	**<.001**	***
Gender	0.039	0.073	0.53	0.594	
LOI (Z-scored)	-0.038	0.012	-3.10	**0.002**	**
Reward x Transition	0.159	0.017	9.62	**<.001**	***
Reward x IQ (Z-scored)	-0.028	0.011	-2.65	**0.008**	**
Reward x Age (Z-scored)	0.050	0.012	4.07	**<.001**	***
Reward x Gender	-0.011	0.024	-0.47	0.641	
Reward x LOI (Z-scored)	-0.019	0.009	-2.10	**0.036**	*
Transition x IQ (Z-scored)	0.006	0.008	0.75	0.455	
Transition x Age (Z-scored)	0.004	0.009	0.42	0.671	
Transition x Gender	0.027	0.016	1.65	0.099	.
Transition x LOI (Z-scored)	-0.009	0.007	-1.19	0.236	
Reward x Transition x IQ (Z-scored)	0.023	0.011	2.20	**0.028**	*
Reward x Transition x Age (Z-scored)	0.021	0.012	1.78	0.075	.
Reward x Transition x Gender	0.075	0.023	3.18	**0.001**	**
Reward x Transition x LOI (Z-scored)	-0.034	0.009	-3.81	**0.000**	***

*p<.05, **p<.01, *** p <.001, SE = Standard Erro

Table S7. Bivariate latent change score model between compulsivity and model-based control

			Estimate	SE	Z-value	P(>|z|)	Std.all
**Latent Variables**							
d Model-based	=~	Model-based (T2)	1.000				1.095
d Compulsivity	=~	Compulsivity (T2)	1.000				1.032
**Regressions**							
Model-based (T2)	~	Model-based (T1)	1.000				1.004
Compulsivity (T2)	~	Compulsivity (T1)	1.000				1.194
d Compulsivity	~	Model-based (T1)	-0.019	0.060	-0.314	0.754	-0.010
d Model-based	~	Compulsivity (T1)	-0.054	0.017	-3.131	0.002	-0.110
d Compulsivity	~	Compulsivity (T1)	-0.524	0.045	-11.657	<0.001	-0.606
d Model-based	~	Model-based (T1)	-0.613	0.039	-15.744	<0.001	-0.562
**Covariances**							
.Model-based (T1)	~~	.Compulsivity (T1)	-0.012	0.004	-2.797	0.005	-0.120
.d Model-based	~~	.d Compulsivity	-0.006	0.002	-2.593	0.010	-0.103
**Intercepts**							
.d Model-based			0.180	0.014	12.504	<0.001	0.783
.Model-based (T1)			0.194	0.009	21.052	<0.001	0.923
.Model-based (T2)			0.000				0.000
.Compulsivity (T2)			0.000				0.000
.d Compulsivity			0.077	0.023	3.307	0.001	0.191
.Compulsivity (T1)			0.451	0.020	22.076	<0.001	0.968
**Variances**							
.Compulsivity (T2)			0.000				0.000
.d Model-based			0.036	0.002	16.775	<0.001	0.687
.Model-based (T1)			0.044	0.003	16.693	<0.001	1.000
.Model-based (T2)			0.000				0.000
.d Compulsivity			0.103	0.011	9.557	<0.001	0.634
.Compulsivity (T1)			0.217	0.023	9.449	<0.001	1.000
**R-Square**			**Estimate**				
Compulsivity (T2)			1.00				
d Model-based			0.313				
Model-based (T2)			1.00				
d Model-based			0.366				

Model corresponding to Figure 3 assessing cross-domain coupling between model-based control and compulsivity. T1, baseline; T2, follow-up; d, estimated latent change score

Table S8. Bivariate latent change score model between compulsivity and model-based control (age, gender and IQ were regressed on observed and latent variables)

			Estimate	SE	Z-value	P(>|z|)	Std.all
**Latent Variables**							
d Model-based	=~	Model-based (T2)	1.000				1.095
d Compulsivity	=~	Compulsivity (T2)	1.000				1.032
**Regressions**							
Model-based (T2)	~	Model-based (T1)	1.000				1.003
Compulsivity (T2)	~	Compulsivity (T1)	1				1.194
d Compulsivity	~	Model-based (T1)	-0.033	0.063	-0.526	0.599	-0.017
d Model-based	~	Compulsivity (T1)	-0.040	0.017	-2.373	0.018	-0.081
d Compulsivity	~	Compulsivity (T1)	-0.533	0.045	-11.813	<0.001	-0.616
d Model-based	~	Model-based (T1)	-0.641	0.040	-15.910	<0.001	-0.588
Model-based (T1)	~	Age	0.093	0.029	3.165	0.002	0.13
d Model-based	~	Age	-0.012	0.030	-0.412	0.680	-0.016
Compulsivity (T1)	~	Age	0.045	0.070	0.646	0.518	0.029
d Compulsivity	~	Age	0.128	0.047	2.728	0.006	0.094
Model-based (T1)	~	Gender	-0.04	0.018	-2.169	0.03	-0.095
d Model-based	~	Gender	-0.015	0.017	-0.914	0.361	-0.033
Compulsivity (T1)	~	Gender	0.041	0.04	1.012	0.312	0.044
d Compulsivity	~	Gender	-0.002	0.029	-0.062	0.95	-0.002
Model-based (T1)	~	IQ	0.029	0.008	3.697	<0.001	0.15
d Model-based	~	IQ	0.034	0.008	4.258	<0.001	0.16
Compulsivity (T1)	~	IQ	-0.08	0.018	-4.583	<0.001	-0.187
d Compulsivity	~	IQ	-0.017	0.014	-1.252	0.211	-0.047
**Covariances**							
.Model-based (T1)	~~	.Compulsivity (T1)	-0.008	0.004	-1.997	0.046	-0.09
.d Model-based	~~	.d Compulsivity	-0.006	0.002	-2.443	0.015	-0.095
Age	~~	IQ	0.038	0.014	2.733	0.006	0.118
Gender	~~	IQ	-0.065	0.023	-2.769	0.006	-0.12
**Intercepts**							
.d Model-based			-0.168	0.101	-1.658	0.097	-0.729
.Model-based (T1)			-0.284	0.099	-2.872	0.004	-1.35
.Model-based (T2)			0.000				0.000
.Compulsivity (T2)			0.000				0.000
.d Compulsivity			0.035	0.172	0.201	0.841	0.086

.Compulsivity (T1)	1.24	0.225	5.517	<0.001	2.663
Age	1.887	0.013	145.28	<0.001	6.371
Gender	0.512	0.022	23.336	<0.001	1.023
IQ	11.133	0.048	233.777	<0.001	10.255
**Variances**					
.Compulsivity (T2)	0.000				0.000
.d Model-based	0.035	0.002	16.738	<0.001	0.661
.Model-based (T1)	0.042	0.003	16.153	<0.001	0.943
.Model-based (T2)	0.000				0.000
.d Compulsivity	0.101	0.011	9.604	<0.001	0.624
.Compulsivity (T1)	0.208	0.022	9.337	<0.001	0.961
Age	0.088	0.004	22.413	<0.001	1
Gender	0.25	0.001	493.944	<0.001	1
IQ	1.178	0.071	16.696	<0.001	1
**R-Square**	**Estimate**				
Compulsivity (T2)	1.00				
d Model-based	0.339				
Model-based (T1)	0.057				
Model-based (T2)	1.000				
d Compulsivity	0.376				
Compulsivity (T1)	0.039				

This model assesses cross-domain coupling between model-based control and compulsivity and includes age, gender and IQ which were regressed both on the observed variables and on the latent change variables of both model-based and compulsivity. The model provided good fit to the data (N = 520; χ_2 = 0.288, df = 1, P = 0.591; RMSEA = 0.000 [0.000, 0.094], SRMR = 0.004, CFI = 1.000, Yuan–Bentler scaling correction factor = 1.001). Results from this model were not numerically nor inferentially different from those reported in Figure S3 and Table S7. T1, baseline; T2, follow-up; d, estimated latent change score.

Table S9. Extended latent change score model between compulsivity, model-based control, and fronto-striatal connectivity

			Estimate	SE	Z-value	P(>|z|)	Std.all
**Latent Variables**							
d Model-based	=~	Model-based (T2)	1.000				1.033
d Compulsivity	=~	Compulsivity (T2)	1.000				0.984
d FC striatum-FPN	=~	FC striatum-FPN (T2)	1.000				1.111
**Regressions**							
Model-based (T2)	~	Model-based (T1)	1.000				0.964
Compulsivity (T2)	~	Compulsivity (T1)	1.000				1.110
FC striatum-FPN (T2)	~	FC striatum-FPN (T1)	1.000				1.003
d Compulsivity	~	Model-based (T1)	-0.083	0.106	-0.781	0.435	-0.045
d Model-based	~	Compulsivity (T1)	-0.083	0.032	-2.603	0.009	-0.159
d FC striatum-FPN	~	Model-based (T1)	0.044	0.053	0.830	0.406	0.052
d FC striatum-FPN	~	Compulsivity (T1)	0.051	0.024	2.107	0.035	0.124
d Compulsivity	~	FC striatum-FPN (T1)	-0.161	0.114	-1.415	0.157	-0.067
d Model-based	~	FC striatum-FPN (T1)	-0.155	0.108	-1.436	0.151	-0.110
d Compulsivity	~	Compulsivity (T1)	-0.497	0.088	-5.616	<0.001	-0.560
d Model-based	~	Model-based (T1)	-0.534	0.070	-7.595	<0.001	-0.498
d FC striatum-FPN	~	FC striatum-FPN (T1)	-0.617	0.070	-8.822	<0.001	-0.557
FC striatum-FPN (T1)	~	Site 1 (T1)	-0.038	0.049	-0.777	0.437	-0.080
FC striatum-FPN (T1)	~	Site 2 (T1)	-0.072	0.044	-1.615	0.106	-0.201
FC striatum-FPN (T2)	~	Site 2 (T2)	-0.035	0.028	-1.221	0.222	-0.076
**Covariances**							
.Model-based (T1)	~~	Compulsivity (T1)	-0.007	0.006	-1.137	0.256	-0.081
. FC striatum-FPN (T1)	~~	Model-based (T1)	0.004	0.002	1.992	0.046	0.137
. FC striatum-FPN (T1)	~~	Compulsivity (T1)	-0.007	0.005	-1.520	0.128	-0.108
.d Model-based	~~	.d Compulsivity	-0.002	0.003	-0.629	0.530	-0.036
.d Model-based	~~	.d FC striatum-FPN	0.000	0.002	0.076	0.939	0.005
.d Compulsivity	~~	.d FC striatum-FPN	0.001	0.003	0.520	0.603	0.032
Site 1 (T1)	~~	Site 1 (T1)	-0.089	0.016	-5.691	<0.001	-0.619
Site 2 (T1)	~~	Site 2 (T2)	0.101	0.016	6.272	<0.001	0.666
Site 1 (T1)	~~	Site 2 (T2)	0.017	0.004	3.973	<0.001	0.149
**Intercepts**							
.d Model-based			0.275	0.054	5.064	<0.001	1.240
.d Compulsivity			0.137	0.067	2.039	0.041	0.364
.d FC striatum-FPN			0.231	0.042	5.539	<0.001	1.323

Model-based (T1)	0.197	0.015	12.763	<0.001	0.949
Compulsivity (T1)	0.404	0.032	12.790	<0.001	0.951
FC striatum-FPN (T1)	0.510	0.042	12.019	<0.001	3.238
Model-based (T2)	0.000				0.000
Compulsivity (T2)	0.000				0.000
FC striatum-FPN (T2)	0.000				0.000
Site 1 (T1)	0.122	0.024	5.004	<0.001	0.372
Site 2 (T1)	0.735	0.033	22.395	<0.001	1.665
Site 2 (T2)	0.862	0.026	33.607	<0.001	2.498
**Variances**					
. Compulsivity (T2)	0.000				0.000
. FC striatum-FPN (T2)	0.000				0.000
. Model-based (T2)	0.000				0.000
.d Model-based	0.035	0.003	10.514	<0.001	0.716
. d Compulsivity	0.098	0.019	5.176	<0.001	0.691
. d FC striatum-FPN	0.020	0.002	8.459	<0.001	0.666
Model-based (T1)	0.043	0.004	10.430	<0.001	1.000
Compulsivity (T1)	0.181	0.034	5.314	<0.001	1.000
FC striatum-FPN (T1)	0.024	0.003	7.660	<0.001	0.973
Site 1 (T1)	0.107	0.018	5.808	<0.001	1.000
Site 2 (T1)	0.195	0.015	12.646	<0.001	1.000
Site 2 (T2)	0.119	0.019	6.414	<0.001	1.000
**R-Square**	**Estimate**				
Compulsivity (T2)	1				
FC striatum-FPN (T2)	1				
Model-based (T2)	1				
d Model-based	0.284				
d Compulsivity	0.309				
D FC striatum-FPN	0.334				
Compulsivity (T1)	0.027				

Model corresponding to Figure 4 assessing cross-domain coupling between model-based control, compulsivity and overall striatal connectivity strength. Site was regressed on functional connectivity measures. T1, baseline; T2, follow-up; d, estimated latent change score

Table S10. Extended latent change score model between compulsivity, model-based control, and fronto-striatal connectivity (age, gender and IQ were regressed on observed and latent variables)

			Estimate	SE	Z-value	P(>|z|)	Std.all
**Latent Variables**							
d Model-based	=~	Model-based (T2)	1.000				1.032
d Compulsivity	=~	Compulsivity (T2)	1.000				0.983
d FC striatum-FPN	=~	FC striatum-FPN (T2)	1.000				1.112
**Regressions**							
Model-based (T2)	~	Model-based (T1)	1				0.957
Compulsivity (T2)	~	Compulsivity (T1)	1				1.111
FC striatum-FPN (T2)	~	FC striatum-FPN (T1)	1				1
d Compulsivity	~	Model-based (T1)	-0.186	0.115	-1.612	0.107	-0.102
d Model-based	~	Compulsivity (T1)	-0.06	0.031	-1.921	0.055	-0.115
d FC striatum-FPN	~	Model-based (T1)	0.028	0.057	0.487	0.627	0.033
d FC striatum-FPN	~	Compulsivity (T1)	0.043	0.025	1.722	0.085	0.104
d Compulsivity	~	FC striatum-FPN (T1)	-0.295	0.127	-2.315	0.021	-0.123
d Model-based	~	FC striatum-FPN (T1)	-0.169	0.106	-1.602	0.109	-0.12
d Compulsivity	~	Compulsivity (T1)	-0.502	0.084	-5.974	<0.001	-0.567
d Model-based	~	Model-based (T1)	-0.574	0.074	-7.764	<0.001	-0.533
d FC striatum-FPN	~	FC striatum-FPN (T1)	-0.652	0.075	-8.644	<0.001	-0.587
FC striatum-FPN (T1)	~	Age	0.096	0.044	2.192	0.028	0.173
d FC striatum-FPN	~	Age	0.061	0.039	1.566	0.117	0.098
Model-based (T1)	~	Age	0.149	0.051	2.918	0.004	0.205
d Model-based	~	Age	-0.038	0.051	-0.743	0.457	-0.048
Compulsivity (T1)	~	Age	-0.076	0.102	-0.75	0.453	-0.051
d Compulsivity	~	Age	0.196	0.075	2.62	0.009	0.147
FC striatum-FPN	~	Gender	-0.078	0.022	-3.545	<0.001	-0.247
d FC striatum-FPN	~	Gender	-0.023	0.023	-1.016	0.31	-0.066
Model-based (T1)	~	Gender	-0.08	0.03	-2.691	0.007	-0.193
d Model-based	~	Gender	-0.02	0.03	-0.68	0.497	-0.045
Compulsivity (T1)	~	Gender	-0.038	0.06	-0.64	0.522	-0.045
d Compulsivity	~	Gender	-0.101	0.052	-1.934	0.053	-0.134
FC striatum-FPN (T1)	~	IQ	-0.002	0.011	-0.18	0.857	-0.014
d FC striatum-FPN	~	IQ	-0.015	0.01	-1.444	0.149	-0.095
Model-based (T1)	~	IQ	0.024	0.012	2.018	0.044	0.132
d Model-based	~	IQ	0.049	0.013	3.612	<0.001	0.244

Compulsivity (T1)	~	IQ	-0.083	0.024	-3.395	0.001	-0.216
d Compulsivity	~	IQ	-0.007	0.016	-0.428	0.668	-0.02
FC striatum-FPN (T1)	~	Site 1 (T1)	-0.016	0.046	-0.358	0.72	-0.034
FC striatum-FPN (T1)	~	Site 2 (T2)	-0.068	0.042	-1.613	0.107	-0.192
FC striatum-FPN (T1)	~	Site 2 (T2)	-0.043	0.029	-1.479	0.139	-0.095
**Covariances**							
.Model-based (T1)	~~	Compulsivity (T1)	-0.004	0.006	-0.6	0.549	-0.045
. FC striatum-FPN (T1)	~~	Model-based (T1)	0.001	0.002	0.53	0.596	0.04
. FC striatum-FPN (T1)	~~	Compulsivity (T1)	-0.007	0.005	-1.439	0.15	-0.112
.d Model-based	~~	.d Compulsivity	-0.002	0.003	-0.638	0.524	-0.037
.d Model-based	~~	.d FC striatum-FPN	0.001	0.002	0.441	0.659	0.031
.d Compulsivity	~~	.d FC striatum-FPN	0	0.003	-0.008	0.994	0
Age	~~	IQ	0.033	0.023	1.412	0.158	0.105
Gender	~~	IQ	-0.059	0.041	-1.427	0.154	-0.105
Site 1 (T1)	~~	Site 2 (T1)	-0.089	0.016	-5.691	<0.001	-0.619
Site 2 (T1)	~~	Site 2 (T2)	0.101	0.016	6.272	<0.001	0.666
Site 1 (T1)	~~	Site 2 (T2)	0.017	0.004	3.973	<0.001	0.149
**Intercepts**							
.d Model-based			-0.180	0.187	-0.961	0.337	-0.810
.d Compulsivity			-0.025	0.212	-0.116	0.908	-0.065
.d FC striatum-FPN			0.322	0.148	2.172	0.030	1.848
Model-based (T1)			-0.319	0.168	-1.893	0.058	-1.547
Compulsivity (T1)			1.485	0.306	4.848	<0.001	3.491
FC striatum-FPN (T1)			0.382	0.135	2.823	0.005	2.436
Model-based (T2)			0.000				0.000
Compulsivity (T2)			0.000				0.000
FC striatum-FPN (T2)			0.000				0.000
Age			1.880	0.021	89.476	<0.001	6.651
Gender			0.470	0.037	12.659	<0.001	0.941
IQ			11.117	0.083	134.256	<0.001	9.994
Site 1 (T1)			0.122	0.024	5.004	<0.001	0.372
Site 2 (T1)			0.735	0.033	22.395	<0.001	1.665
Site 2 (T2)			0.862	0.026	33.607	<0.001	2.498
**Variances**							
. Compulsivity (T2)			0.000				0.000
. FC striatum-FPN (T2)			0.000				0.000
. Model-based (T2)			0.000				0.000

.d Model-based	0.032	0.003	10.852	<0.001	0.658
. d Compulsivity	0.093	0.017	5.497	<0.001	0.657
. d FC striatum-FPN	0.020	0.002	8.879	<0.001	0.647
Model-based (T1)	0.038	0.004	9.782	<0.001	0.892
Compulsivity (T1)	0.172	0.033	5.281	<0.001	0.948
FC striatum-FPN (T1)	0.022	0.003	8.123	<0.001	0.880
Age	0.080	0.006	12.785	<0.001	1.000
Gender	0.249	0.002	110.482	<0.001	1.000
IQ	1.237	0.127	9.737	<0.001	1.000
Site 1 (T1)	0.107	0.018	5.808	<0.001	1.000
Site 2 (T1)	0.195	0.015	12.646	<0.001	1.000
Site 2 (T2)	0.119	0.019	6.414	<0.001	1.000
**R-Square**	**Estimate**				
Compulsivity (T2)	1				
FC striatum-FPN (T2)	1				
Model-based (T2)	1				
d Model-based	0.342				
d Compulsivity	0.343				
d FC striatum-FPN	0.353				
Model-based (T1)	0.108				
Compulsivity (T1)	0.052				
FC striatum-FPN (T1)	0.12				

This model assesses cross-domain coupling between model-based control, compulsivity and overall striatal connectivity. The model includes not only site, but also age, gender and IQ which were regressed both on the observed variables and on the latent change variables of model-based, compulsivity and overall striatal connectivity. (Model fit: N = 181; χ_2 = 58.443, df = 25, P = <0.001; RMSEA = 0.086[0.051, 0.121], SRMR = 0.050, CFI = 0.971, Yuan–Bentler scaling correction factor = 0.686). T1, baseline; T2, follow-up; d, estimated latent change score.

Table S11. List of cortical regions selected based on frontoparietal mask from Yeo et al., 2011

N	Nomenclature (Glasser et al., 2016) for areas included in FPN	
1	L_7Pm_ROI	Superior Parietal Cortex
2	L_8C_ROI	Dorsolateral Prefrontal Cortex
3	L_IFJa_ROI	Inferior Frontal Cortex
4	L_IFSp_ROI	Inferior Frontal Cortex
5	L_IFSa_ROI	Inferior Frontal Cortex
6	L_p9-46v_ROI	Dorsolateral Prefrontal Cortex
7	L_46_ROI	Dorsolateral Prefrontal Cortex
8	L_a9-46v_ROI	Dorsolateral Prefrontal Cortex
9	L_9-46d_ROI	Dorsolateral Prefrontal Cortex
10	L_11l_ROI	Orbital and Polar Frontal Cortex
11	L_i6-8_ROI	Dorsolateral Prefrontal Cortex
12	L_AVI_ROI	Insular and Frontal Opercular Cortex
13	L_IP2_ROI	Inferior Parietal Cortex
14	L_IP1_ROI	Inferior Parietal Cortex
15	L_p47r_ROI	Inferior Frontal Cortex
16	L_a32pr_ROI	Anterior cingulate and Medial Prefrontal Cortex
17	R_POS2_ROI	Posterior Cingulate Cortex
18	R_7Pm_ROI	Superior Parietal Cortex
19	R_8BM_ROI	Anterior cingulate and Medial Prefrontal Cortex
20	R_8Av_ROI	Dorsolateral Prefrontal Cortex
21	R_8C_ROI	Dorsolateral Prefrontal Cortex
22	R_44_ROI	Inferior Frontal Cortex
23	R_a47r_ROI	Orbital and Polar Frontal Cortex
24	R_IFJa_ROI	Inferior Frontal Cortex
25	R_IFSp_ROI	Inferior Frontal Cortex
26	R_IFSa_ROI	Inferior Frontal Cortex
27	R_p9-46v_ROI	Dorsolateral Prefrontal Cortex
28	R_46_ROI	Dorsolateral Prefrontal Cortex
29	R_a9-46v_ROI	Dorsolateral Prefrontal Cortex
30	R_9-46d_ROI	Dorsolateral Prefrontal Cortex
31	R_a10p_ROI	Orbital and Polar Frontal Cortex
32	R_11l_ROI	Orbital and Polar Frontal Cortex
33	R_i6-8_ROI	Dorsolateral Prefrontal Cortex
34	R_s6-8_ROI	Dorsolateral Prefrontal Cortex
35	R_AVI_ROI	Insular and Frontal Opercular Cortex
36	R_TE1p_ROI	Lateral Temporal Cortex
37	R_IP2_ROI	Inferior Parietal Cortex
38	R_IP1_ROI	Inferior Parietal Cortex
39	R_PFm_ROI	Inferior Parietal Cortex
40	R_p10p_ROI	Orbital and Polar Frontal Cortex
41	R_p47r_ROI	Inferior Frontal Cortex
42	R_a32pr_ROI	Anterior cingulate and Medial Prefrontal Cortex

Table S12. Neuroscience in Psychiatry Network (NSPN) consortium author list

**NSPN Principal investigators**	Edward Bullmore<br>Raymond Dolan<br>Ian Goodyer<br>Peter Fonagy<br>Peter Jones
**NSPN (funded) staff**	Michael Moutoussis<br>Tobias Hauser<br>Sharon Neufeld<br>Rafael Romero-Garcia<br>Michelle St Clair<br>Petra Vértes<br>Kirstie Whitaker<br>Becky Inkster<br>Gita Prabhu<br>Cinly Ooi<br>Umar Toseeb<br>Barry Widmer<br>Junaid Bhatti<br>Laura Villis<br>Ayesha Alrumaithi<br>Sarah Birt<br>Aislinn Bowler<br>Kalia Cleridou<br>Hina Dadabhoy<br>Emma Davies<br>Ashlyn Firkins<br>Sian Granville<br>Elizabeth Harding<br>Alexandra Hopkins<br>Daniel Isaacs<br>Janchai King<br>Danae Kokorikou<br>Christina Maurice<br>Cleo McIntosh<br>Jessica Memarzia<br>Harriet Mills<br>Ciara O'Donnell<br>Sara Pantaleone<br>Jenny Scott<br>Andrea Reiter<br>Lucy Vanes

Figure S1. Stability and convergent validity of compulsivity questionnaire

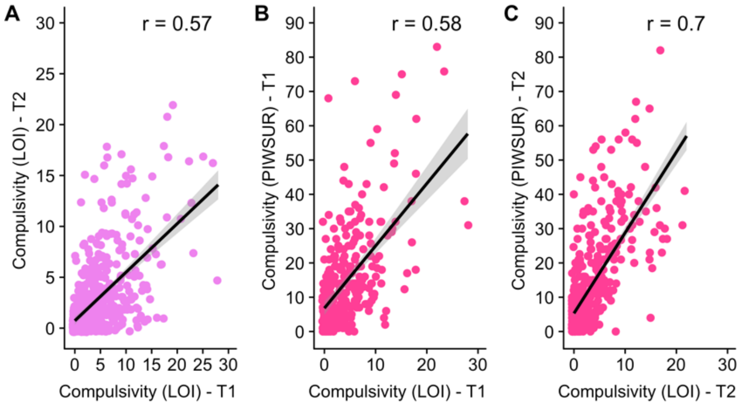

In our sample, the questionnaire to measure compulsivity which was available on most subjects both at baseline (T1) and followup (T2) was the Leyton Obsessional Inventory-Child Version Survey (LOI, N = 520; T1: mean ± std = 4.51 ± 4.66, range = 0-28, T2: mean ± std = 2.88 ± 3.9, range = 0-22, Bramber et al., 2002). Therefore, we made use of this measure for our main analyses. This measure proved moderately reliable (A). In addition, both at baseline (N=277) (B) and followup (N=519) (C), LOI showed convergent validity with another questionnaire in the battery also meant to measure compulsivity, namely the Padua Inventory Washington State University Revision (PI-WSUR, T1: mean ±std = 15.04±15.05, range = 0 - 83.5; T2: mean ± std = 12.17±13.63, range = 0 - 99 Burns et al., 1996). Overall, our results suggest that our results pertain to compulsivity. Pearson's correlation coefficients are provided in panels A-C.

Figure S2. Display of cortical brain regions included within cortical frontoparietal mask

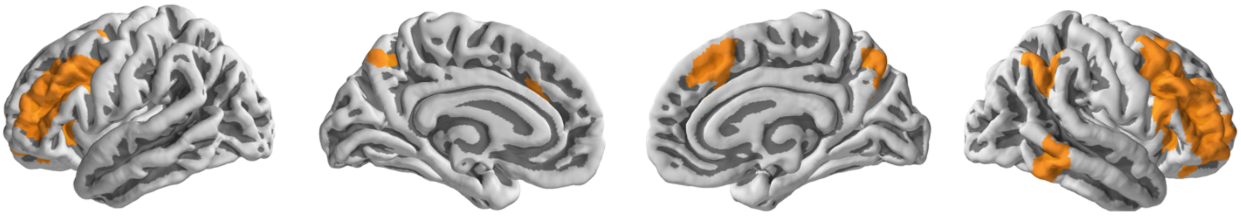

Figure S3. Regional effects of compulsivity on development of fronto-striatal connectivity

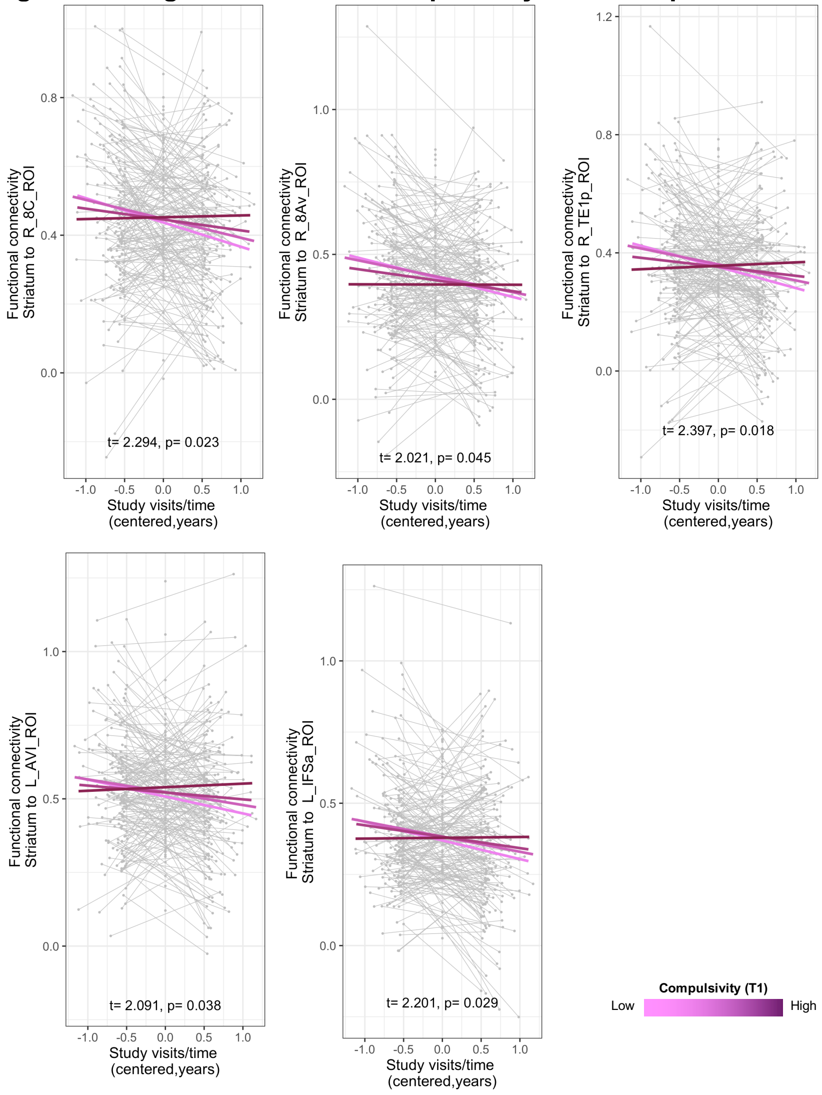

Longitudinal developmental changes in fronto-striatal functional connectivity are reduced in subjects with high compulsivity. Longitudinal models for functional connectivity where compulsivity scores at baseline (T1) were used to predict rate of change in connectivity between the striatum and each of the cortical regions included in the FPN. For illustration purposes only, the subjects were divided into 4 groups based on 1st, 2nd, 3rd and 4th quantile of the distribution of compulsivity at baseline. Lines illustrate model's compulsivity by visits/time interaction suggesting dependent rate of fronto-striatal connectivity over study visits for different levels of compulsivity (i.e., compulsivity x visits/time interaction $P < 0.05$, uncorrected for multiple comparisons; individual t and p-values are shown in each panel). Pink to purple coloration for lower to higher compulsivity respectively. The slowing in functional changes is seen mostly in regions comprising portions of the dorsolateral prefrontal cortex, inferior frontal gyrus and anterior insula. 8Av,8C Dorsolateral prefrontal cortex; TE1p Auditory Cortex; AVI Anterior Ventral Insular Area; IFSa Inferior frontal cortex. Nomenclature refers to Glasser et al., 2016. R, right; L, left. Regions for which a significant interaction between compulsivity and time was found are shown in the panels. These findings indicate that early in adolescence, high compulsivity is linked to relative lack of change in functional connectivity within fronto-striatal circuits of known importance for the pathological manifestation of OCD and related disorders (N= 408 scans; 230 subjects).

Figure S4. Overall and regional effects of compulsivity using a composite compulsivity measure

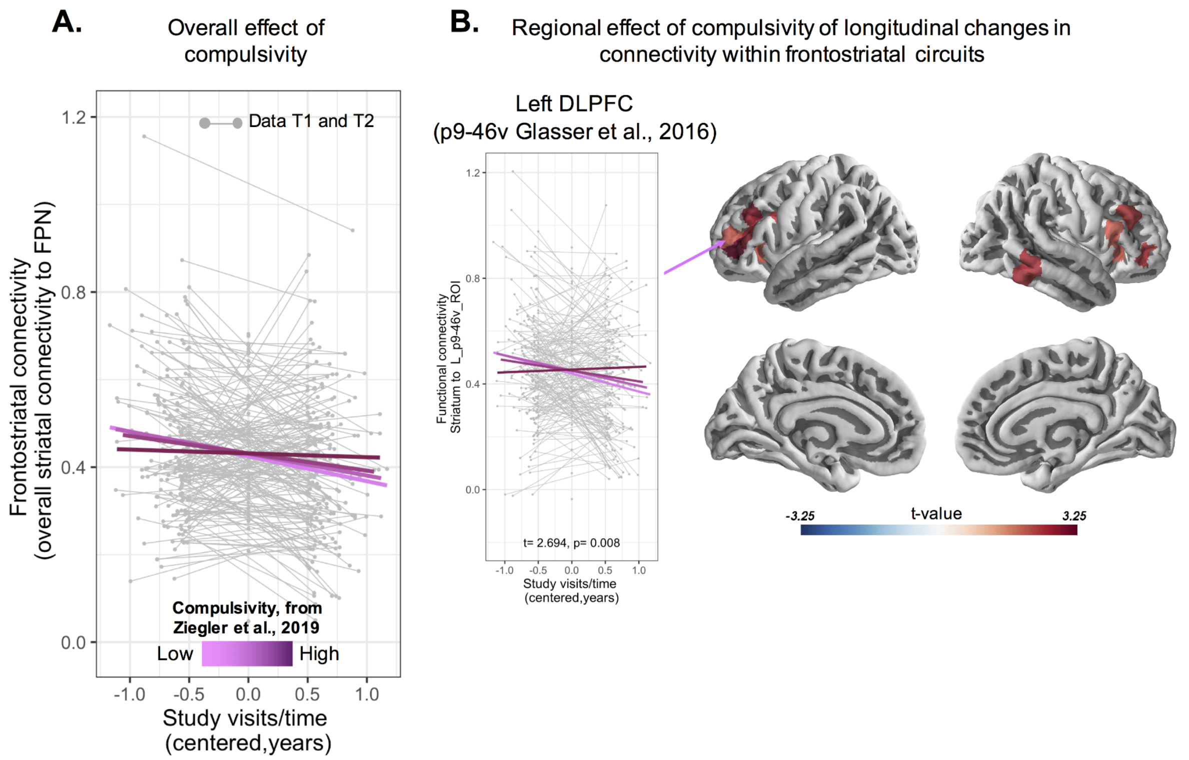

Longitudinal developmental changes in fronto-striatal functional connectivity are reduced in subjects with high compulsivity. A. Longitudinal model for functional connectivity where compulsivity scores were used to predict rate of change in connectivity between the striatum and the FPN network. This analysis is analogous to the one reported in the main paper (Figure 5and Figure S3), but a different compulsivity score was used. To test robustness of our findings, we used here a compulsivity measure similar to the one used by Ziegler and colleagues (2019) which investigated longitudinal changes in myelin marker as a function of compulsivity scores derived from principal component analysis. We identified a significant compulsivity by time interaction (β= 0.016, SE = 0.01, t = 2.19, df = 178; P =0.030) on the overall striatal nodal strength to the FPN. **B.** We investigated regional specificity of these changes. The slowing in functional changes is seen in regions overlapping with those identified in the main manuscript and, with regions where compulsivity has been independently shown to affects myelin growth (Ziegler et al., 2019). The panel shows a thresholded statistical map of regions for which an interaction between compulsivity at T1 and visits/time was observed at $p < 0.05$ (uncorrected for multiple comparisons).

Figure S5. Effects of head motion on variables of interest

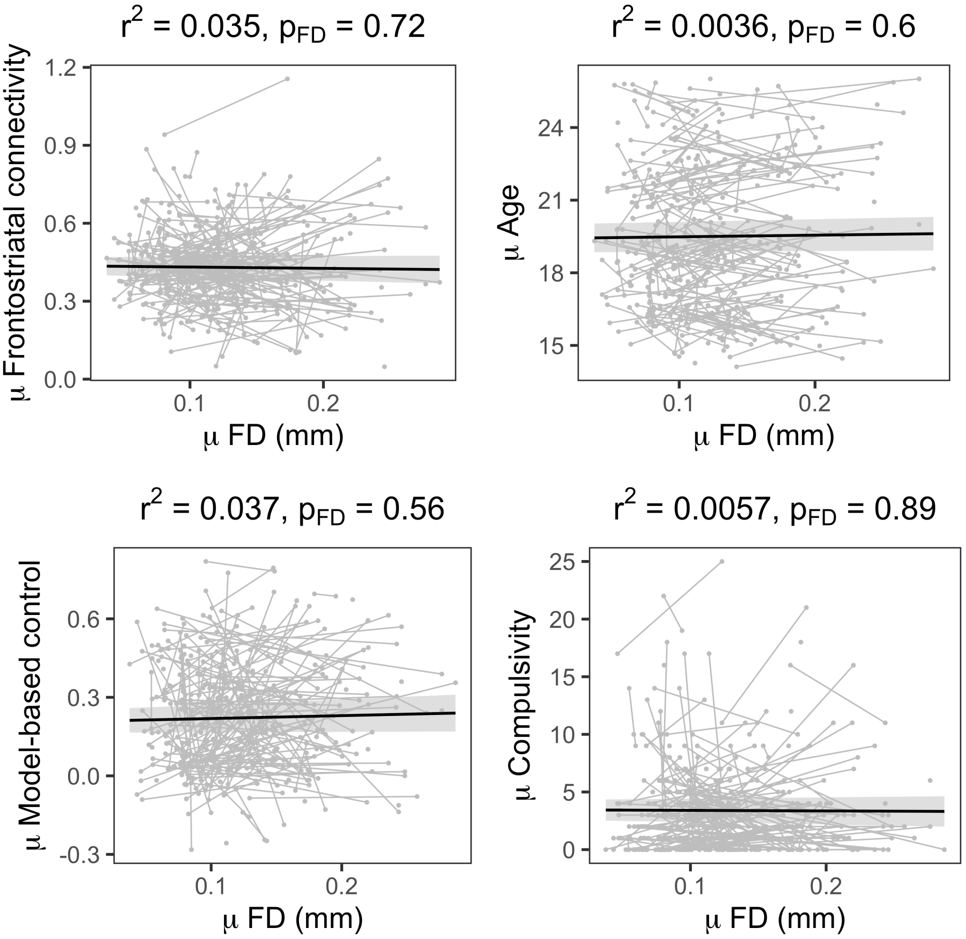

Head motion is not significantly associated with variables of interest. We investigated the relationship between head motion (quantified as mean FD) and each variable of interest. As described in the main text, effects of head movement on connectivity were corrected by regressing functional connectivity on mean FD; the residuals constitute participant-specific FD corrected functional connectivity. We qualified the relationship between FD and any variable of interest in this study. There was no significant association between FD and overall striatal connectivity, age, model-based control, nor compulsivity. For each figure the pseudo r^2 for GLMMs includes variance in mean functional connectivity, mean age, mean model-based control, or mean compulsivity explained by mean FD, as well as gender.

Supplementary References

1. B. Kiddle, *et al.*, Cohort Profile: The NSPN 2400 Cohort: a developmental sample supporting the Wellcome Trust NeuroScience in Psychiatry Network. *Int. J. Epidemiol.* **47**, 18–19g (2018).

2. N. D. Daw, S. J. Gershman, B. Seymour, P. Dayan, R. J. Dolan, Model-based influences on humans' choices and striatal prediction errors. *Neuron* **69**, 1204–1215 (2011).

3. N. Shahar, *et al.*, Credit assignment to state-independent task representations and its relationship with model-based decision making. *Proc. Natl. Acad. Sci.*, 201821647 (2019).

4. A. R. Otto, C. M. Raio, A. Chiang, E. A. Phelps, N. D. Daw, Working-memory capacity protects model-based learning from stress. *Proc. Natl. Acad. Sci. U. S. A.* **110**, 20941–20946 (2013).

5. G. L. Burns, S. G. Keortge, G. M. Formea, L. G. Sternberger, Revision of the Padua Inventory of obsessive compulsive disorder symptoms: distinctions between worry, obsessions, and compulsions. *Behav. Res. Ther.* **34**, 163–173 (1996).

6. E. B. Foa, *et al.*, The obsessive-compulsive inventory: development and validation of a short version. *Psychol. Assess.* **14**, 485–496 (2002).

7. F. Váša, *et al.*, Conservative and disruptive modes of adolescent change in human brain functional connectivity. *Proc. Natl. Acad. Sci.* **117**, 3248–3253 (2020).

8. B. Fischl, M. I. Sereno, A. M. Dale, Cortical Surface-Based Analysis: II: Inflation, Flattening, and a Surface-Based Coordinate System. *NeuroImage* **9**, 195–207 (1999).

9. F. Váša, *et al.*, Adolescent Tuning of Association Cortex in Human Structural Brain Networks. *Cereb. Cortex N. Y. N 1991* **28**, 281–294 (2018).

10. K. J. Whitaker, *et al.*, Adolescence is associated with genomically patterned consolidation of the hubs of the human brain connectome. *Proc. Natl. Acad. Sci.* **113**, 9105–9110 (2016).

11. R. W. Cox, AFNI: software for analysis and visualization of functional magnetic resonance neuroimages. *Comput. Biomed. Res. Int. J.* **29**, 162–173 (1996).

12. Z. S. Saad, *et al.*, A new method for improving functional-to-structural MRI alignment using local Pearson correlation. *NeuroImage* **44**, 839–848 (2009).

13. P. Kundu, *et al.*, Integrated strategy for improving functional connectivity mapping using multiecho fMRI. *Proc. Natl. Acad. Sci.*, 201301725 (2013).

14. P. Kundu, S. J. Inati, J. W. Evans, W.-M. Luh, P. A. Bandettini, Differentiating BOLD and non-BOLD signals in fMRI time series using multi-echo EPI. *NeuroImage* **60**, 1759–1770 (2012).

15. A. X. Patel, *et al.*, A wavelet method for modeling and despiking motion artifacts from resting-state fMRI time series. *NeuroImage* **95**, 287–304 (2014).

16. J. D. Power, K. A. Barnes, A. Z. Snyder, B. L. Schlaggar, S. E. Petersen, Spurious but systematic correlations in functional connectivity MRI networks arise from subject motion. *NeuroImage* **59**, 2142–2154 (2012).

17. T. D. Satterthwaite, *et al.*, Motion artifact in studies of functional connectivity: Characteristics and mitigation strategies. *Hum. Brain Mapp.* **40**, 2033–2051 (2019).

18. S. Gu, *et al.*, Emergence of system roles in normative neurodevelopment. *Proc. Natl. Acad. Sci.* **112**, 13681–13686 (2015).

19. G. Ziegler, *et al.*, Compulsivity and impulsivity traits linked to attenuated developmental frontostriatal myelination trajectories. *Nat. Neurosci.* **22**, 992–999 (2019).

20. B. Guillaume, *et al.*, Fast and accurate modelling of longitudinal and repeated measures neuroimaging data. *NeuroImage* **94**, 287–302 (2014).
